# Supplementary material for: Spatial variation in socio-economic vulnerability to Influenza-like Infection for the US population
Source: PLoS Comput Biol. 2026 Jan 28;22(1):e1013839. doi: 10.1371/journal.pcbi.1013839 (PMC12919934; doi:10.1371/journal.pcbi.1013839)
Supplement: S1 Table — (DOCX) [file pcbi.1013839.s001.docx]

| **Variable** | **Justification** | **References** |
| --- | --- | --- |
| Income in the past 12 months below poverty level | Overcrowded living conditions; Limited access to health care; Limited health literacy | (Keene & Blankenship, 2023; Mazenda & Lubinga, 2024; Scarpino et al., 2020) |
| Household received Food Stamps/SNAP in the past 12 months | Lower income, Limited access to healthcare and understanding of health information, Poorer nutrition and housing conditions, Job requires exposure to larger groups | (Berkowitz et al., 2017; Carlson & Llobrera, 2022) |
| Income $100,000 or more | 21% less chance of getting flu vaccine, better living conditions, Access to health care | (C. M. Gaskin et al., 2023) |
| Population Density | Increased Contact Rates; Higher levels of socioeconomic inequality; Crowded common places | (Kianoush et al., 2022; Tunnicliffe & Warren-Gash, 2022; Yin et al., 2021) |
| Total Population | Easier transmission; Higher levels of socioeconomic inequality | (Hartfield & Alizon, 2013; Zhang et al., 2021) |
| Percent of Female | Hormonal differences; Sex differences in response to vaccines; Humoral immune system | (Flanagan et al., 2017; Giurgea et al., 2022; Halsey et al., 2013; Klein et al., 2012) |
| Moved from abroad | Low Vaccination rate; Delayed/ limited health care access; Occupational and living conditions; Language barrier; Fear of Deportation in undocumented immigrants | (Daniels et al., 2022; Hunter-Adams & Rother, 2017; Khullar & Chokshi, 2019; Nanakali et al., 2023) |
| 65 years and over | Reduced immunity; Delayed health care access; Improper hygiene and nutrition | (McGovern et al., 2024; Møgelmose et al., 2023; Or et al., 2020; Rodriguez et al., 2021) |
| Population 25 years and over- Less than 9th grade | Lower educational attainment often indicates lower income, Limited access to healthcare, Poor nutrition and housing conditions | (Montez & Cheng, 2022; Raghupathi & Raghupathi, 2020; Zajacova & Lawrence, 2018) |
| Population 65 years and over- High school graduate or higher | Increased health literacy; Better economic situation | (Brunello et al., 2016; Hoenig & Wenz, 2021) |
| No computer | Limiting access to information and healthcare support, Low health literacy; Lack of awareness; Indicator of lower income group | (Johnson & Hariharan, 2017; Levy & and Janke, 2016; Shahid et al., 2022) |
| Without an Internet subscription | Limiting access to information and healthcare support, Low health literacy; Lack of awareness; indicator of lower income group | (Bashshur et al., 2020; Duplaga, 2021; Yu & Meng, 2022) |
| Mean travel time to work (minutes) | Increased exposure time; crowded mode of transportation; Stress and fatigue; Higher concentrations of people with lower socioeconomic status | (Han et al., 2023; Lachapelle & Boisjoly, 2023; White et al., 2019) |
| Median earnings (dollars) | Limited Access to Healthcare; Poor Living Conditions | ( Christeon M Gaskin et al., 2022; Guo et al., 2022; Lazar & and Davenport, 2018; O’Donnell, 2024) |
| Fraction population in owner occupied | Stronger community ties and social networks; Long term houses; No overcrowding | (Ahmad et al., 2020; Chan et al., 2015) |
| Housing Median value (dollars) | Indicator of economic condition | (Grewal et al., 2024; Jin et al., 2024) |
| White | Higher Vaccination rates; Better access to healthcare | (Black et al., 2009; Breaux & Rooks, 2022; Yearby et al., 2022) |
| Black or African American | Certain underlying health conditions; Socioeconomic disparities | (Kodsup & Godebo, 2023; Macias-Konstantopoulos et al., 2023; Millett et al., 2020) |
| American Indian and Alaska Native | Socioeconomic disadvantages; Higher rates of chronic health conditions; Racism and historical trauma | (Doxey et al., 2019) |
| Asian | Poverty, Limited access to healthcare, Lack of education; Social interaction; Housing conditions; Pre-existing genetic susceptibility | OMH U.S. Department of Health and Human services, Office of minority health (Davidson et al., 2021a; Obra et al., 2021; Srivastav et al., 2018; Yom & Lor, 2022) |
| Native Hawaiian and Other Pacific Islander | Disparities in healthcare access, economic stability, and chronic disease prevalence; Systemic barriers, including cultural and language differences, can hinder effective outreach and healthcare delivery; Reducing vaccination rates and timely medical care | OMH U.S. Department of Health and Human services, Office of minority health (Davidson et al., 2021b; Muramatsu & Chin, 2024) |
| Some Other Race | Disparities in healthcare access, economic stability, and chronic disease prevalence; Systemic barriers, including cultural and language differences, can hinder effective outreach and healthcare delivery; Reducing vaccination rates and timely medical care | (Chandran & Schulman, 2022) |
| Hispanic Population | Low vaccination rates; Language barriers, Limited healthcare access | (Cabral & Cuevas, 2020; Fisher et al., 2023) |
| With a vision difficulty | Low vaccination rates; Limited healthcare access | (Onukwugha et al., 2024; Varadaraj et al., 2022) |
| With a cognitive difficulty | Low vaccination rates; Limited healthcare access | (Lovett et al., 2023) |
| With an ambulatory difficulty | Limited access to healthcare and vaccination; Dependency on Assistance; Difficulty Following Preventive Measures | (Okoro et al., 2018) |
| With a self-care difficulty | Limited access to healthcare and vaccination; Dependency on Assistance; Difficulty Following Preventive Measures | (Martínez et al., 2021) |
| With an independent living difficulty | Limited access to healthcare and vaccination; Dependency on Assistance; Difficulty Following Preventive Measures | (DiGennaro Reed et al., 2014; Long et al., 2023) |
| Percent of 65 and above with health insurance coverage | More access to healthcare; Seek early treatment and vaccination; Treatment of underlying chronic conditions | (Akpalu et al., 2020; Barker & Li, 2020; McGovern et al., 2024) |
| Percentage Native born- No Insurance | More likely economically challenged; Limited access to healthcare; Unmanaged underlying conditions | (Akpalu et al., 2020; Kumar et al., 2014) |
| Percentage Foreign Born- Naturalized- No Insurance | Lower vaccination rate; Historical susceptibility; Limited health care access | (Kraut, 2010; Truman et al., 2009) |
| Percentage Foreign Born- Noncitizen- No Insurance | Limited access to healthcare; lower income and language barriers; Fear of Deportation in undocumented immigrants | (Dunajcik & Cunningham, 2023; Singh et al., 2013) |
| Adults any disability | Limited access to healthcare; Lower income and language barriers; | (Castro et al., 2023) |
| Asthma among adults | Compromised Respiratory System; Compromised immunity; | (Veerapandian et al., 2018) |
| Chronic obstructive pulmonary disease among adults | Weakened Respiratory System; Suppressed the immune system | (Bhat et al., 2015; Wu et al., 2023) |
| Diabetes among adults | Weakened Immune System; Medication Interactions | (Dicembrini et al., 2023; Thomas et al., 2022) |
| Influenza vaccination among adults | Reduced transmission and symptoms | (Olson et al., 2022; Trombetta et al., 2022) |
| Invasive cancer incidence (per 100000) | Weakened Immune System; Medication Interactions; frequent hospitalizations | (Hajjar et al., 2010; Li et al., 2020) |
| Pneumococcal vaccination among adults aged 65 years and older | Reflects Healthcare Access; Indicator of Preventive Health Behavior | (Liu, Kelly D., et al., 2023; Liu, Linda, et al., 2023) |

**References**

1. Ahmad, K., Erqou, S., Shah, N., Nazir, U., Morrison, A. R., Choudhary, G., & Wu, W.-C. (2020). Association of poor housing conditions with COVID-19 incidence and mortality across US counties. *PLOS ONE*, *15*(11), e0241327-. https://doi.org/10.1371/journal.pone.0241327
2. Akpalu, Y., Sullivan, S. J., & Regan, A. K. (2020). Association between health insurance coverage and uptake of seasonal influenza vaccine in Brazos County, Texas. *Vaccine*, *38*(9), 2132–2135. https://doi.org/https://doi.org/10.1016/j.vaccine.2020.01.029
3. Barker, A. R., & Li, L. (2020). The cumulative impact of health insurance on health status. *Health Services Research*, *55*(S2), 815–822. https://doi.org/https://doi.org/10.1111/1475-6773.13325
4. Bashshur, R., Doarn, C. R., Frenk, J. M., Kvedar, J. C., & Woolliscroft, J. O. (2020). Telemedicine and the COVID-19 Pandemic, Lessons for the Future. *Telemedicine and E-Health*, *26*(5), 571–573. https://doi.org/10.1089/tmj.2020.29040.rb
5. Berkowitz, S. A., Seligman, H. K., Rigdon, J., Meigs, J. B., & Basu, S. (2017). Supplemental Nutrition Assistance Program (SNAP) participation and health care expenditures among low-income adults. *JAMA Internal Medicine*, *177*(11), 1642–1649. https://doi.org/10.1001/jamainternmed.2017.4841
6. Bhat, T. A., Panzica, L., Kalathil, S. G., & Thanavala, Y. (2015). Immune Dysfunction in Patients with Chronic Obstructive Pulmonary Disease. *Annals of the American Thoracic Society*, *12*(Supplement 2), S169–S175. https://doi.org/10.1513/AnnalsATS.201503-126AW
7. Black, C. L., Hung, M.-C., Srivastav, A., Lu, P., Garg, S., Jhung, M., Fry, A., Jatlaoui, T. C., Davenport, E., & Burns, E. (2009). *Vital Signs: Influenza Hospitalizations and Vaccination Coverage by Race and Ethnicity—United States, 2009–10 Through 2021–22 Influenza Seasons*. https://www.cdc.gov/nchs/nvss/bridged_race.htm
8. Breaux, R. D., & Rooks, R. N. (2022). The intersectional importance of race/ethnicity, disability, and age in flu vaccine uptake for U.S. adults. *SSM - Population Health*, *19*, 101211. https://doi.org/https://doi.org/10.1016/j.ssmph.2022.101211
9. Brunello, G., Fort, M., Schneeweis, N., & Winter-Ebmer, R. (2016). The Causal Effect of Education on Health: What is the Role of Health Behaviors? *Health Economics*, *25*(3), 314–336. https://doi.org/https://doi.org/10.1002/hec.3141
10. Cabral, J., & Cuevas, A. G. (2020). Health Inequities Among Latinos/Hispanics: Documentation Status as a Determinant of Health. *Journal of Racial and Ethnic Health Disparities*, *7*(5), 874–879. https://doi.org/10.1007/s40615-020-00710-0
11. Carlson, S., & Llobrera, J. (2022). *SNAP Is Linked with Improved Health Outcomes and Lower Health Care Costs*. https://www.cbpp.org/research/food-assistance/the-supplemental-nutrition-assistance-program-snap
12. Castro, F. F., Varadaraj, V., Reed, N. S., & Swenor, B. K. (2023). Disparities in influenza vaccination for U.S. adults with disabilities living in community settings by race/ethnicity, 2016–2021. *Disability and Health Journal*, *16*(3), 101477. https://doi.org/https://doi.org/10.1016/j.dhjo.2023.101477
13. Chan, D. P. C., Wong, N. S., Wong, E. L. Y., Cheung, A. W. L., & Lee, S. S. (2015). Household characteristics and influenza vaccination uptake in the community-dwelling elderly: a cross-sectional study. *Preventive Medicine Reports*, *2*, 803–808. https://doi.org/https://doi.org/10.1016/j.pmedr.2015.09.002
14. Chandran, M., & Schulman, K. A. (2022). Racial disparities in healthcare and health. *Health Services Research*, *57*(2), 218–222. https://doi.org/https://doi.org/10.1111/1475-6773.13957
15. Daniels, D., Imdad, A., Buscemi-Kimmins, T., Vitale, D., Rani, U., Darabaner, E., Shaw, A., & Shaw, J. (2022). Vaccine hesitancy in the refugee, immigrant, and migrant population in the United States: A systematic review and meta-analysis. In *Human Vaccines and Immunotherapeutics* (Vol. 18, Issue 6). Taylor and Francis Ltd. https://doi.org/10.1080/21645515.2022.2131168
16. Davidson, J., Banerjee, A., Mathur, R., Ramsay, M., Smeeth, L., Walker, J., McDonald, H., & Warren-Gash, C. (2021a). Ethnic differences in the incidence of clinically diagnosed influenza: an England population-based cohort study 2008-2018 [version 3; peer review: 2 approved]. *Wellcome Open Research*, *6*(49). https://doi.org/10.12688/wellcomeopenres.16620.3
17. Davidson, J., Banerjee, A., Mathur, R., Ramsay, M., Smeeth, L., Walker, J., McDonald, H., & Warren-Gash, C. (2021b). Ethnic differences in the incidence of clinically diagnosed influenza: an England population-based cohort study 2008-2018 [version 3; peer review: 2 approved]. *Wellcome Open Research*, *6*(49). https://doi.org/10.12688/wellcomeopenres.16620.3
18. Dicembrini, I., Silverii, G. A., Clerico, A., Fornengo, R., Gabutti, G., Sordi, V., Tafuri, S., Peruzzi, O., & Mannucci, E. (2023). Influenza: Diabetes as a risk factor for severe related-outcomes and the effectiveness of vaccination in diabetic population. A meta-analysis of observational studies. *Nutrition, Metabolism and Cardiovascular Diseases*, *33*(6), 1099–1110. https://doi.org/https://doi.org/10.1016/j.numecd.2023.03.016
19. DiGennaro Reed, F. D., Strouse, M. C., Jenkins, S. R., Price, J., Henley, A. J., & Hirst, J. M. (2014). Barriers to Independent Living for Individuals with Disabilities and Seniors. *Behavior Analysis in Practice*, *7*(2), 70–77. https://doi.org/10.1007/s40617-014-0011-6
20. Doxey, M., Chrzaszcz, L., Dominguez, A., & James, R. D. (2019). A Forgotten Danger: Burden of Influenza Mortality Among American Indians and Alaska Natives, 1999-2016. *Journal of Public Health Management and Practice*, *25*. https://journals.lww.com/jphmp/fulltext/2019/09001/a_forgotten_danger__burden_of_influenza_mortality.4.aspx
21. Dunajcik, A., & Cunningham, S. A. (2023). On the basis of visa type: Insights into incorporation and health among foreign-born people in the United States. *Journal of Migration and Health*, *7*, 100146. https://doi.org/https://doi.org/10.1016/j.jmh.2022.100146
22. Duplaga, M. (2021). The association between Internet use and health-related outcomes in older adults and the elderly: a cross-sectional study. *BMC Medical Informatics and Decision Making*, *21*(1), 150. https://doi.org/10.1186/s12911-021-01500-2
23. Fisher, C., Bragard, E., & Madhivanan, P. (2023). COVID-19 Vaccine Hesitancy among Economically Marginalized Hispanic Parents of Children under Five Years in the United States. *Vaccines*, *11*(3). https://doi.org/10.3390/vaccines11030599
24. Flanagan, K. L., Fink, A. L., Plebanski, M., & Klein, S. L. (2017). Sex and Gender Differences in the Outcomes of Vaccination over the Life Course. *Annual Review of Cell and Developmental Biology*, *33*(Volume 33, 2017), 577–599. https://doi.org/https://doi.org/10.1146/annurev-cellbio-100616-060718
25. Gaskin, C. M., Woods, D. R., Ghosh, S., Watson, S., & Huber, L. R. (2023). The Effect of Income Disparities on Influenza Vaccination Coverage in the United States. *Public Health Reports*, *138*(1), 85–90. https://doi.org/10.1177/00333549211069190
26. Gaskin, Christeon M, Woods, Darien R, Ghosh, Subhanwita, Watson, Shae, & Huber, Larissa R. (2022). The Effect of Income Disparities on Influenza Vaccination Coverage in the United States. *Public Health Reports®*, *138*(1), 85–90. https://doi.org/10.1177/00333549211069190
27. Giurgea, L. T., Cervantes-Medina, A., Walters, K.-A., Scherler, K., Han, A., Czajkowski, L. M., Baus, H. A., Hunsberger, S., Klein, S. L., Kash, J. C., Taubenberger, J. K., & Memoli, M. J. (2022). Sex Differences in Influenza: The Challenge Study Experience. *The Journal of Infectious Diseases*, *225*(4), 715–722. https://doi.org/10.1093/infdis/jiab422
28. Grewal, A., Hepburn, K. J., Lear, S. A., Adshade, M., & Card, K. G. (2024). The impact of housing prices on residents’ health: a systematic review. *BMC Public Health*, *24*(1), 931. https://doi.org/10.1186/s12889-024-18360-w
29. Guo, J., Hernandez, I., Dickson, S., Tang, S., Essien, U. R., Mair, C., & Berenbrok, L. A. (2022). Income disparities in driving distance to health care infrastructure in the United States: a geographic information systems analysis. *BMC Research Notes*, *15*(1), 225. https://doi.org/10.1186/s13104-022-06117-w
30. Hajjar, L. A., Mauad, T., Galas, F. R. B. G., Kumar, A., da Silva, L. F. F., Dolhnikoff, M., Trielli, T., Almeida, J. P., Borsato, M. R. L., Abdalla, E., Pierrot, L., Kalil Filho, R., Auler, J. O. C., Saldiva, P. H. N., & Hoff, P. M. (2010). Severe novel influenza A (H1N1) infection in cancer patients. *Annals of Oncology*, *21*(12), 2333–2341. https://doi.org/https://doi.org/10.1093/annonc/mdq254
31. Halsey, N. A., Griffioen, M., Dreskin, S. C., Dekker, C. L., Wood, R., Sharma, D., Jones, J. F., LaRussa, P. S., Garner, J., Berger, M., Proveaux, T., Vellozzi, C., Broder, K., Setse, R., Pahud, B., Hrncir, D., Choi, H., Sparks, R., Williams, S. E., … Kelso, J. M. (2013). Immediate hypersensitivity reactions following monovalent 2009 pandemic influenza A (H1N1) vaccines: Reports to VAERS. *Vaccine*, *31*(51), 6107–6112. https://doi.org/10.1016/j.vaccine.2013.09.066
32. Han, L., Peng, C., & Xu, Z. (2023). The Effect of Commuting Time on Quality of Life: Evidence from China. *International Journal of Environmental Research and Public Health*, *20*(1). https://doi.org/10.3390/ijerph20010573
33. Hartfield, M., & Alizon, S. (2013). Introducing the Outbreak Threshold in Epidemiology. *PLoS Pathogens*, *9*(6). https://doi.org/10.1371/journal.ppat.1003277
34. Hoenig, K., & Wenz, S. E. (2021). Education, health behavior, and working conditions during the pandemic: evidence from a German sample. *European Societies*, *23*(S1), S275–S288. https://doi.org/10.1080/14616696.2020.1824004
35. Hunter-Adams, J., & Rother, H. A. (2017). A Qualitative study of language barriers between South African health care providers and cross-border migrants. *BMC Health Services Research*, *17*(1), 1–9. https://doi.org/10.1186/s12913-017-2042-5
36. Jin, S., Zheng, H., Marantz, N., & Roy, A. (2024). Understanding the effects of socioeconomic factors on housing price appreciation using explainable AI. *Applied Geography*, *169*, 103339. https://doi.org/https://doi.org/10.1016/j.apgeog.2024.103339
37. Johnson, E. J., & Hariharan, S. (2017). Public health awareness: knowledge, attitude and behaviour of the general public on health risks during the H1N1 influenza pandemic. *Journal of Public Health*, *25*(3), 333–337. https://doi.org/10.1007/s10389-017-0790-7
38. Keene, D. E., & Blankenship, K. M. (2023). The Affordable Rental Housing Crisis and Population Health Equity: a Multidimensional and Multilevel Framework. In *Journal of Urban Health* (Vol. 100, Issue 6, pp. 1212–1223). Springer. https://doi.org/10.1007/s11524-023-00799-8
39. Khullar, D., & Chokshi, D. A. (2019). Challenges for immigrant health in the USA&#x2014; the road to crisis. *The Lancet*, *393*(10186), 2168–2174. https://doi.org/10.1016/S0140-6736(19)30035-2
40. Kianoush, S., Rifai, M. Al, Patel, J., Michos, E. D., Alam, M., Samad, Z., Sohail, R., Cader, F. A., Sayed, A., Zhu, D., & Virani, S. S. (2022). Racial Disparity in Flu Vaccine Uptake Among Asian American Individuals: A National Health Interview Survey Study. *Current Problems in Cardiology*, *47*(12), 101391. https://doi.org/https://doi.org/10.1016/j.cpcardiol.2022.101391
41. Klein, S. L., Hodgson, A., & Robinson, D. P. (2012). Mechanisms of sex disparities in influenza pathogenesis. *Journal of Leukocyte Biology*, *92*(1), 67–73. https://doi.org/10.1189/jlb.0811427
42. Kodsup, P., & Godebo, T. R. (2023). Disparities in Underlying Health Conditions and COVID-19 Infection and Mortality in Louisiana, USA. *Journal of Racial and Ethnic Health Disparities*, *10*(2), 805–816. https://doi.org/10.1007/s40615-022-01268-9
43. Kraut,  Alan M. (2010). Immigration, Ethnicity, and the Pandemic. *Public Health Reports®*, *125*(3_suppl), 123–133. https://doi.org/10.1177/00333549101250S315
44. Kumar, G., Taneja, A., Majumdar, T., Jacobs, E. R., Whittle, J., Nanchal, R., & Investigators, from the M. I. in C. C. O. R. (MICCOR) G. of. (2014). The Association of Lacking Insurance with Outcomes of Severe Sepsis: Retrospective Analysis of an Administrative Database*. *Critical Care Medicine*, *42*(3). https://journals.lww.com/ccmjournal/fulltext/2014/03000/the_association_of_lacking_insurance_with_outcomes.11.aspx
45. Lachapelle, U., & Boisjoly, G. (2023). Breaking down public transit travel time for more accurate transport equity policies: A trip component approach. *Transportation Research Part A: Policy and Practice*, *175*, 103756. https://doi.org/https://doi.org/10.1016/j.tra.2023.103756
46. Lazar, M., & and Davenport, L. (2018). Barriers to Health Care Access for Low Income Families: A Review of Literature. *Journal of Community Health Nursing*, *35*(1), 28–37. https://doi.org/10.1080/07370016.2018.1404832
47. Levy, H., & and Janke, A. (2016). Health Literacy and Access to Care. *Journal of Health Communication*, *21*(sup1), 43–50. https://doi.org/10.1080/10810730.2015.1131776
48. Li, J., Zhang, D., Sun, Z., Bai, C., & Zhao, L. (2020). Influenza in hospitalised patients with malignancy: a propensity score matching analysis. *ESMO Open*, *5*(5), e000968. https://doi.org/https://doi.org/10.1136/esmoopen-2020-000968
49. Liu, J., Kelly D., J., & and Shoener Dunham, L. (2023). Pneumococcal vaccination coverage among US adults enrolled in Medicaid and newly diagnosed with underlying medical conditions. *Expert Review of Vaccines*, *22*(1), 596–607. https://doi.org/10.1080/14760584.2023.2226718
50. Liu, J., Linda, S. D., & and Johnson, K. D. (2023). Regional factors associated with pneumococcal vaccination coverage among U.S. adults with underlying chronic or immunocompromising conditions. *Human Vaccines & Immunotherapeutics*, *19*(1), 2194779. https://doi.org/10.1080/21645515.2023.2194779
51. Long, C., Plenn, E., Acri, S., & Richardson, C. (2023). The Impact of Living Situation on Healthcare Encounters for Individuals with Intellectual Disability. *Cureus*. https://doi.org/10.7759/cureus.51156
52. Lovett, Rebecca M, Benavente, Julia Yoshino, Opsasnick, Lauren A, Weiner-Light, Sophia, Curtis,  Laura M, & Wolf, Michael S. (2023). Associations Between Cognitive Impairment Severity and Barriers to Healthcare Engagement Among Older Adults. *Journal of Applied Gerontology*, *42*(7), 1387–1396. https://doi.org/10.1177/07334648231166289
53. Macias-Konstantopoulos, W. L., Collins, K. A., Diaz, R., Duber, H. C., Edwards, C. D., Hsu, A. P., Ranney, M. L., Riviello, R. J., Wettstein, Z. S., & Sachs, C. J. (2023). Race, Healthcare, and Health Disparities: A Critical Review and Recommendations for Advancing Health Equity. *Western Journal of Emergency Medicine*, *24*(5), 906–918. https://doi.org/10.5811/WESTJEM.58408
54. Martínez, N., Connelly, C. D., Pérez, A., & Calero, P. (2021). Self-care: A concept analysis. *International Journal of Nursing Sciences*, *8*(4), 418–425. https://doi.org/https://doi.org/10.1016/j.ijnss.2021.08.007
55. Mazenda, A., & Lubinga, M. (2024). Healthcare access and deprivation in low-income urban households. *Discover Social Science and Health*, *4*(1). https://doi.org/10.1007/s44155-024-00108-x
56. McGovern, I., Cappell, K., Bogdanov, A. N., & Haag, M. D. M. (2024). Incidence of Influenza-related Medical Encounters and the Associated Healthcare Resource Use and Complications Across Adult Age Groups in the United States During the 2015–2020 Influenza Seasons. *Clinical Infectious Diseases*, *79*(3), 778–786. https://doi.org/10.1093/cid/ciae180
57. Millett, G. A., Jones, A. T., Benkeser, D., Baral, S., Mercer, L., Beyrer, C., Honermann, B., Lankiewicz, E., Mena, L., Crowley, J. S., Sherwood, J., & Sullivan, P. S. (2020). Assessing differential impacts of COVID-19 on black communities. *Annals of Epidemiology*, *47*, 37–44. https://doi.org/https://doi.org/10.1016/j.annepidem.2020.05.003
58. Møgelmose, S., Neels, K., Beutels, P., & Hens, N. (2023). Exploring the impact of population ageing on the spread of emerging respiratory infections and the associated burden of mortality. *BMC Infectious Diseases*, *23*(1). https://doi.org/10.1186/s12879-023-08657-3
59. Montez, J. K., & Cheng, K. J. (2022). Educational disparities in adult health across U.S. states: Larger disparities reflect economic factors. *Frontiers in Public Health*, *10*. https://doi.org/10.3389/fpubh.2022.966434
60. Muramatsu, N., & Chin, M. H. (2024). Asian, Native Hawaiian, and Pacific Islander Populations in the US—Moving From Invisibility to Health Equity. *JAMA Network Open*, *7*(5), e2411617–e2411617. https://doi.org/10.1001/jamanetworkopen.2024.11617
61. Nanakali, S. S., Hassan, O., Silva, L., Al-Oraibi, A., Chaloner, J., Gogoi, M., Qureshi, I., Sahare, P., Pareek, M., Chattopadhyay, K., & Nellums, L. B. (2023). Migrants’ living conditions, perceived health needs and implications for the use of antibiotics and antimicrobial resistance in the United Kingdom: A qualitative study. *Health Science Reports*, *6*(10), e1655. https://doi.org/https://doi.org/10.1002/hsr2.1655
62. Obra, J. K., Lin, B., Đoàn, L. N., Palaniappan, L., & Srinivasan, M. (2021). Achieving Equity in Asian American Health Care: Critical Issues and Solutions. In *Journal of Asian Health* (Vol. 13).
63. O’Donnell, O. (2024). Health and health system effects on poverty: A narrative review of global evidence. *Health Policy*, *142*, 105018. https://doi.org/https://doi.org/10.1016/j.healthpol.2024.105018
64. Okoro, C. A., Hollis, N. D., Cyrus, A. C., & Griffin-Blake, S. (2018). *Morbidity and Mortality Weekly Report Prevalence of Disabilities and Health Care Access by Disability Status and Type Among Adults-United States, 2016*. https://aspe.hhs.gov/datacncl/standards/aca/4302/index.pdf.
65. Olson, S. M., Newhams, M. M., Halasa, N. B., Feldstein, L. R., Novak, T., Weiss, S. L., Coates, B. M., Schuster, J. E., Schwarz, A. J., Maddux, A. B., Hall, M. W., Nofziger, R. A., Flori, H. R., Gertz, S. J., Kong, M., Sanders Jr, R. C., Irby, K., Hume, J. R., Cullimore, M. L., … Investigators, P. I. C. I. (2022). Vaccine Effectiveness Against Life-Threatening Influenza Illness in US Children. *Clinical Infectious Diseases*, *75*(2), 230–238. https://doi.org/10.1093/cid/ciab931
66. Onukwugha, C., Castro, F., Swenor, B. K., & Varadaraj, V. (2024). Disparities in Healthcare Access for Adults with Self-Reported Vision Difficulty - Behavioral Risk Factor Surveillance System 2019–2021. *Ophthalmic Epidemiology*, *0*(0), 1–6. https://doi.org/10.1080/09286586.2024.2407905
67. Or, P. P.-L., Wong, B. Y.-M., & Chung, J. W.-Y. (2020). To investigate the association between the health literacy and hand hygiene practices of the older adults to help them fight against infectious diseases in Hong Kong. *American Journal of Infection Control*, *48*(5), 485–489. https://doi.org/https://doi.org/10.1016/j.ajic.2019.12.021
68. Raghupathi, V., & Raghupathi, W. (2020). The influence of education on health: An empirical assessment of OECD countries for the period 1995-2015. *Archives of Public Health*, *78*(1). https://doi.org/10.1186/s13690-020-00402-5
69. Rodriguez, I. J., Lalinde Ruiz, N., Llano León, M., Martínez Enríquez, L., Montilla Velásquez, M. del P., Ortiz Aguirre, J. P., Rodríguez Bohórquez, O. M., Velandia Vargas, E. A., Hernández, E. D., & Parra López, C. A. (2021). Immunosenescence Study of T Cells: A Systematic Review. *Frontiers in Immunology*, *11*. https://doi.org/10.3389/fimmu.2020.604591
70. Scarpino, S. V., Scott, J. G., Eggo, R. M., Clements, B., Dimitrov, N. B., & Meyers, L. A. (2020). Socioeconomic bias in influenza surveillance. *PLoS Computational Biology*, *16*(7). https://doi.org/10.1371/journal.pcbi.1007941
71. Shahid, R., Shoker, M., Chu, L. M., Frehlick, R., Ward, H., & Pahwa, P. (2022). Impact of low health literacy on patients’ health outcomes: a multicenter cohort study. *BMC Health Services Research*, *22*(1), 1148. https://doi.org/10.1186/s12913-022-08527-9
72. Singh, G. K., Rodriguez-Lainz, A., & Kogan, M. D. (2013). Immigrant Health Inequalities in the United States: Use of Eight Major National Data Systems. *The Scientific World Journal*, *2013*(1), 512313. https://doi.org/https://doi.org/10.1155/2013/512313
73. Srivastav, A., O’Halloran, A., Lu, P.-J., & Williams, W. W. (2018). Influenza Vaccination Coverage Among English-Speaking Asian Americans. *American Journal of Preventive Medicine*, *55*(5), e123–e137. https://doi.org/https://doi.org/10.1016/j.amepre.2018.06.018
74. Thomas, S., Ouhtit, A., Al Khatib, H. A., Eid, A. H., Mathew, S., Nasrallah, G. K., Emara, M. M., Al Maslamani, M. A., & Yassine, H. M. (2022). Burden and disease pathogenesis of influenza and other respiratory viruses in diabetic patients. *Journal of Infection and Public Health*, *15*(4), 412–424. https://doi.org/https://doi.org/10.1016/j.jiph.2022.03.002
75. Trombetta, C. M., Kistner, O., Montomoli, E., Viviani, S., & Marchi, S. (2022). Influenza Viruses and Vaccines: The Role of Vaccine Effectiveness Studies for Evaluation of the Benefits of Influenza Vaccines. In *Vaccines* (Vol. 10, Issue 5). MDPI. https://doi.org/10.3390/vaccines10050714
76. Truman, B. I., Tinker, T., Vaughan, E., Kapella, B. K., Brenden, M., Woznica, C. V, Rios, E., & Lichtveld, M. (2009). Pandemic Influenza Preparedness and Response Among Immigrants and Refugees. *American Journal of Public Health*, *99*(S2), S278–S286. https://doi.org/10.2105/AJPH.2008.154054
77. Tunnicliffe, L., & Warren-Gash, C. (2022). Investigating the effects of population density of residence and rural/urban classification on rate of influenza-like illness symptoms in England and Wales. *Influenza and Other Respiratory Viruses*, *16*(6), 1183–1190. https://doi.org/10.1111/irv.13032
78. Varadaraj, V., Ehrlich, J. R., & Swenor, B. K. (2022). Vision Impairment Has Implications for Aging and Health Outcomes, Beyond Ophthalmology. *JAMA Network Open*, *5*(5), e2214610–e2214610. https://doi.org/10.1001/jamanetworkopen.2022.14610
79. Veerapandian, R., Snyder, J. D., & Samarasinghe, A. E. (2018). Influenza in asthmatics: For better or for worse? In *Frontiers in Immunology* (Vol. 9, Issue AUG). Frontiers Media S.A. https://doi.org/10.3389/fimmu.2018.01843
80. White, M. J., Yin, H. S., Rothman, R. L., Sanders, L. M., Delamater, A., Flower, K., & Perrin, E. M. (2019). Neighborhood Commute to Work Times and Self-Reported Caregiver Health Behaviors and Food Access. *Academic Pediatrics*, *19*(1), 74–79. https://doi.org/https://doi.org/10.1016/j.acap.2018.07.005
81. Wu, W., Li, Z., Wang, Y., Huang, C., Zhang, T., & Zhao, H. (2023). Advances in metabolomics of chronic obstructive pulmonary disease. *Chinese Medical Journal Pulmonary and Critical Care Medicine*, *1*(4), 223–230. https://doi.org/https://doi.org/10.1016/j.pccm.2023.10.001
82. Yearby, R., Clark, B., & Figueroa, J. F. (2022). Structural Racism In Historical And Modern US Health Care Policy. *Health Affairs*, *41*(2), 187–194. https://doi.org/10.1377/hlthaff.2021.01466
83. Yin, H., Sun, T., Yao, L., Jiao, Y., Ma, L., Lin, L., Carolyn Graff, J., Aleya, L., Postlethwaite, A., Gu, W., & Chen, H. (2021). *Association between population density and infection rate suggests the importance of social distancing and travel restriction in reducing the COVID-19 pandemic*. https://doi.org/10.1007/s11356-021-12364-4/Published
84. Yom, S., & Lor, M. (2022). Advancing Health Disparities Research: The Need to Include Asian American Subgroup Populations. *Journal of Racial and Ethnic Health Disparities*, *9*(6), 2248–2282. https://doi.org/10.1007/s40615-021-01164-8
85. Yu, J., & Meng, S. (2022). Impacts of the Internet on Health Inequality and Healthcare Access: A Cross-Country Study. *Frontiers in Public Health*, *10*. https://doi.org/10.3389/fpubh.2022.935608
86. Zajacova, A., & Lawrence, E. M. (2018). The Relationship Between Education and Health: Reducing Disparities Through a Contextual Approach. *Annual Review of Public Health*, *39*(Volume 39, 2018), 273–289. https://doi.org/10.1146/annurev-publhealth-031816-044628
87. Zhang, X., Liu, H., Tang, H., Zhang, M., Yuan, X., & Shen, X. (2021). The effect of population size for pathogen transmission on prediction of COVID-19 spread. *Scientific Reports*, *11*(1). https://doi.org/10.1038/s41598-021-97578-9
