# Supplementary material for: Spatial variation in socio-economic vulnerability to Influenza-like Infection for the US population
Source: PLoS Comput Biol. 2026 Jan 28;22(1):e1013839. doi: 10.1371/journal.pcbi.1013839 (PMC12919934; doi:10.1371/journal.pcbi.1013839)
Supplement: S2 Table — (DOCX) [file pcbi.1013839.s002.docx]

| **Variable** | **Direction**  **(+1: Positive and -1: Negative)** |
| --- | --- |
| Population density | +1: Positive |
| Percent Black or African American | +1: Positive |
| Percent American Indian and Alaska Native | +1: Positive |
| Percent Native Hawaiian and Other Pacific Islander | -1: Negative |
| Percent Female | +1: Positive |
| Percent Moved from abroad | +1: Positive |
| Percent pop (> 65) | +1: Positive |
| Percent Household received Food Stamps/SNAP (12 months) | +1: Positive |
| Fraction population in owner occupied | -1: Negative |
| Percent with health insurance coverage (> 65) | +1: Positive |
| No computer | +1: Positive |
| Mean travel time to work (minutes) | -1: Negative |
| Percent with a cognitive difficulty | +1: Positive |
| Percent Native born No Insurance | -1: Negative |
| Percent Foreign Born Noncitizen No Insurance | -1: Negative |
| Asthma among adults | -1: Negative |
| Diabetes among adults | -1: Negative |
| Influenza vaccination among adults | +1: Positive |
| Invasive cancer incidence | -1: Negative |
| Pneumococcal vaccination (>65) | +1: Positive |
| Percent Hispanic | +1: Positive |
| Total Population | -1: Negative |
