# Supplementary figures and images for: Spatial variation in socio-economic vulnerability to Influenza-like Infection for the US population
Source: PLoS Comput Biol. 2026 Jan 28;22(1):e1013839. doi: 10.1371/journal.pcbi.1013839 (PMC12919934; doi:10.1371/journal.pcbi.1013839)

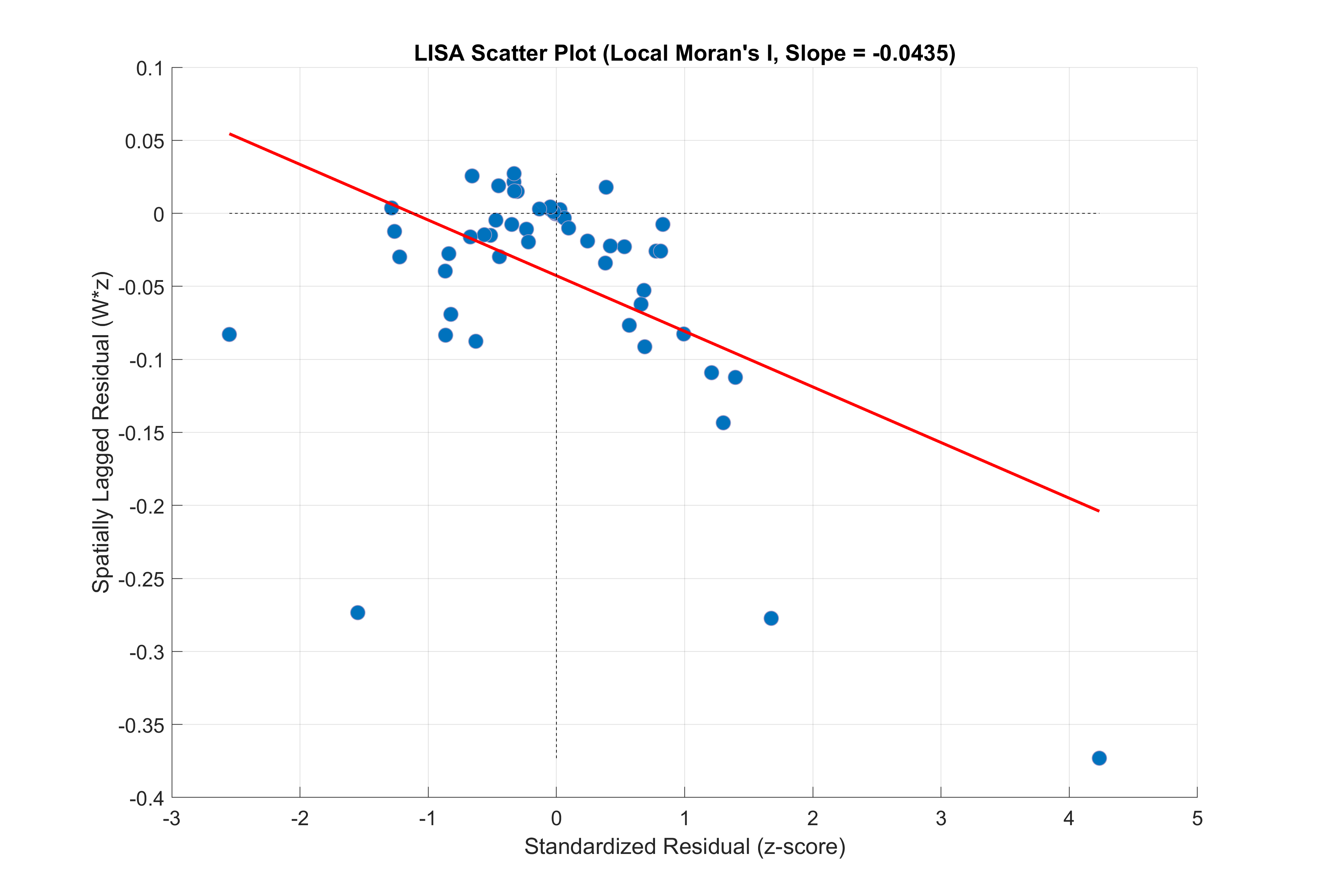

Supplement: S1 Fig — The negative slope (Moran’s I = –0.0435, p = 0.0041) indicates significant spatial dispersion, suggesting that neighboring states tend to exhibit dissimilar residual values, with no evidence of spatial clustering. (PNG) [file pcbi.1013839.s003.png]

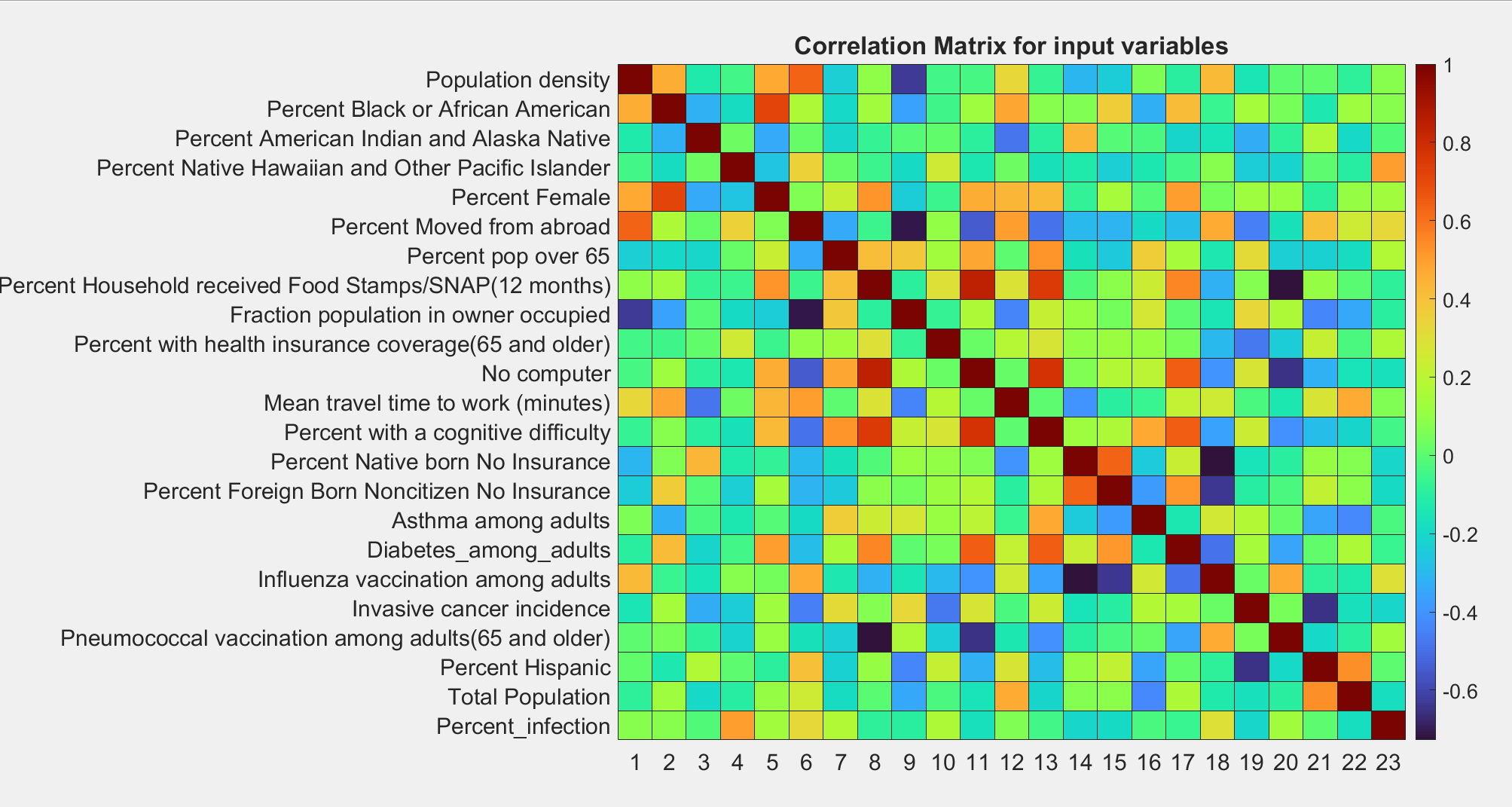

Supplement: S2 Fig — (TIF) [file pcbi.1013839.s004.tif]

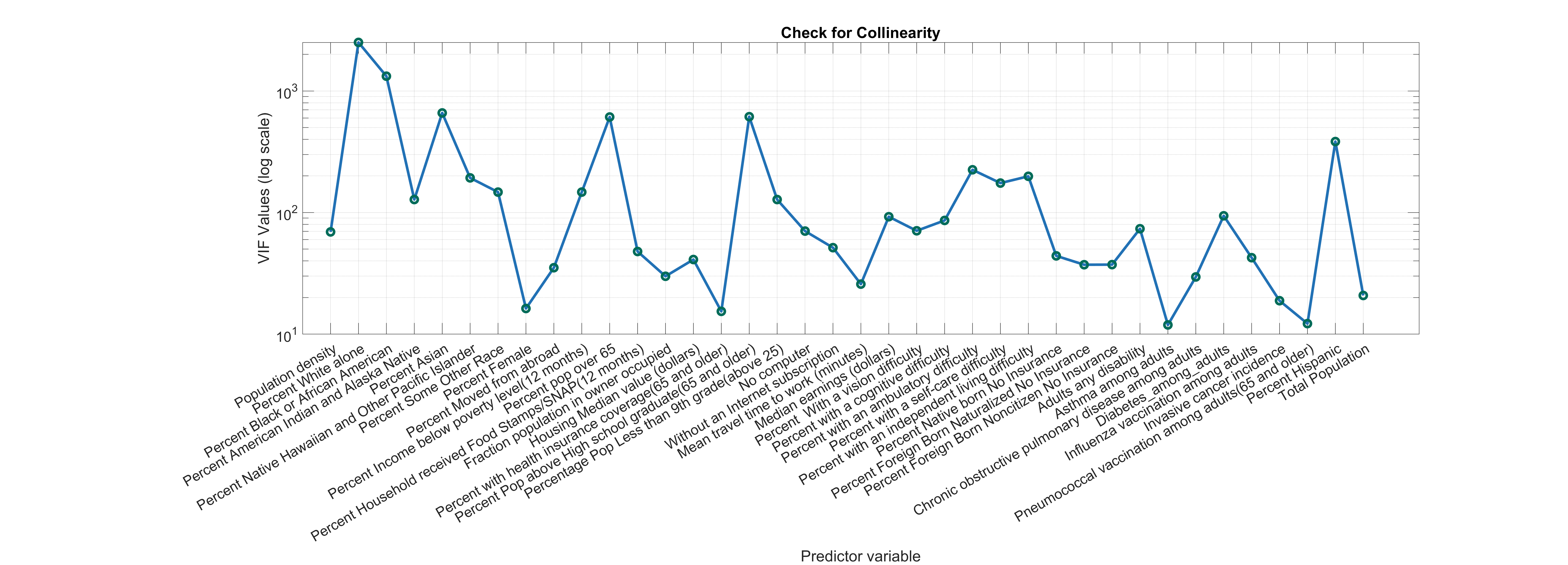

Supplement: S3 Fig — (TIF) [file pcbi.1013839.s005.tif]

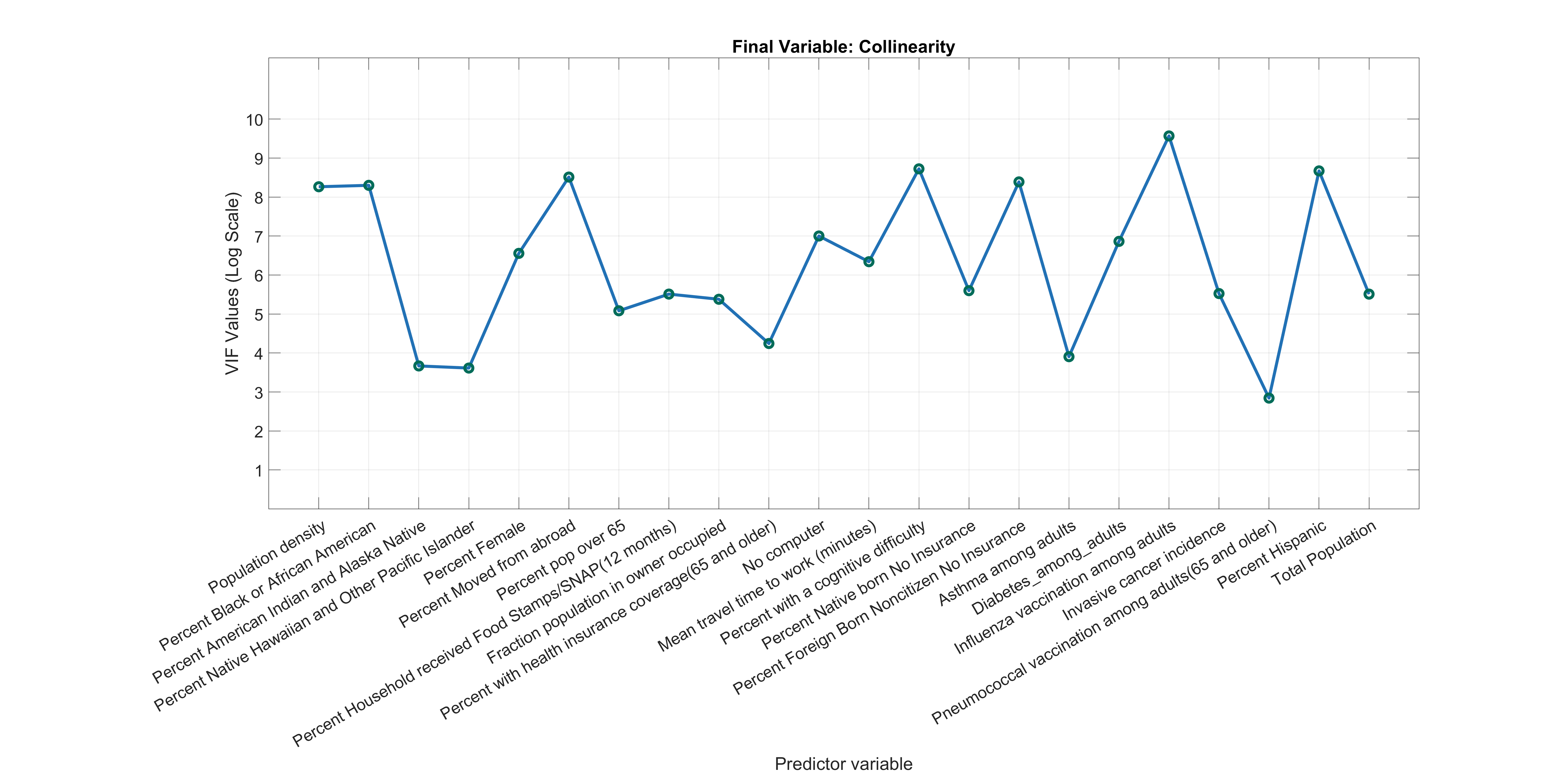

Supplement: S4 Fig — All the variables have values less than 10. (TIF) [file pcbi.1013839.s006.tif]

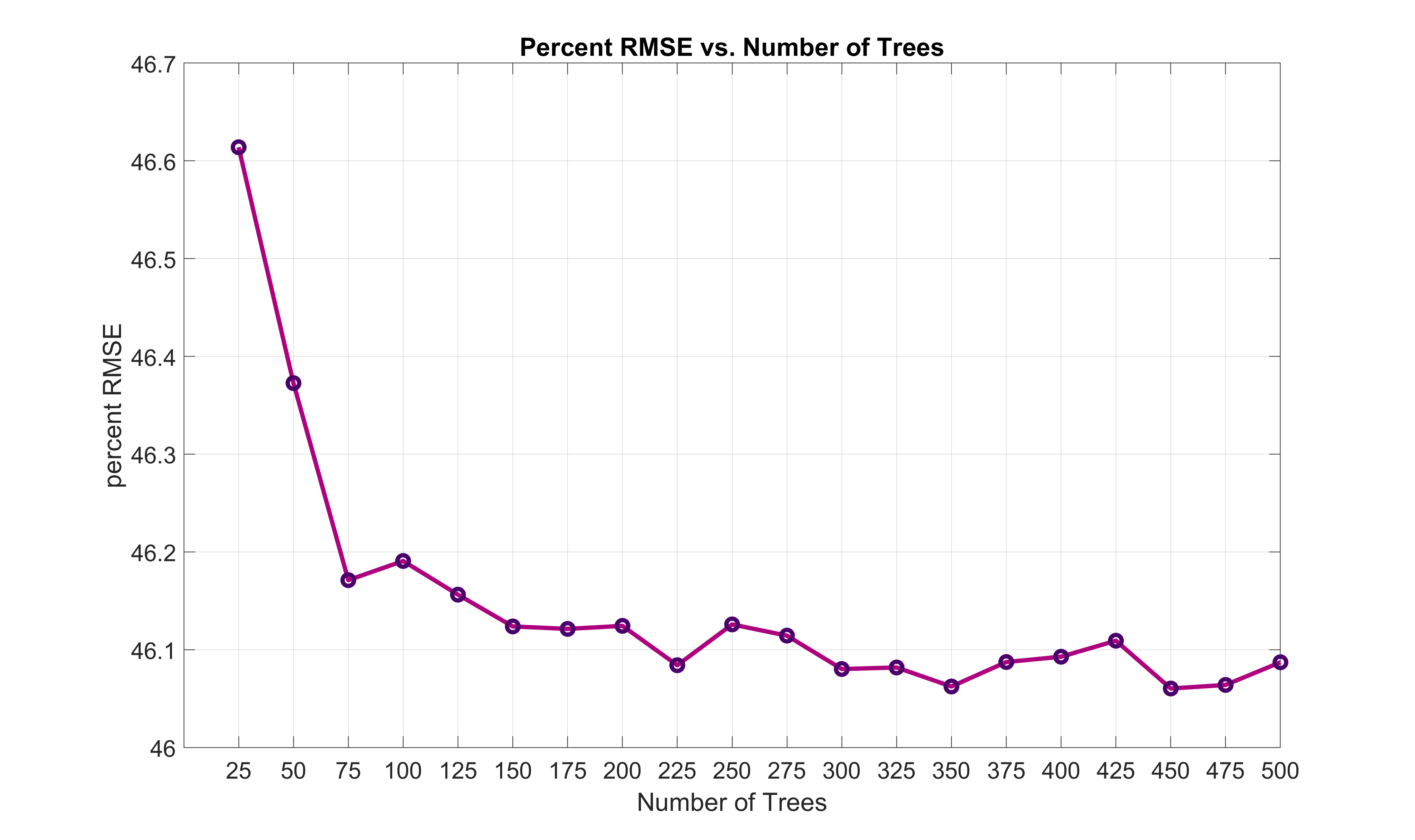

Supplement: S5 Fig — (TIF) [file pcbi.1013839.s007.tif]

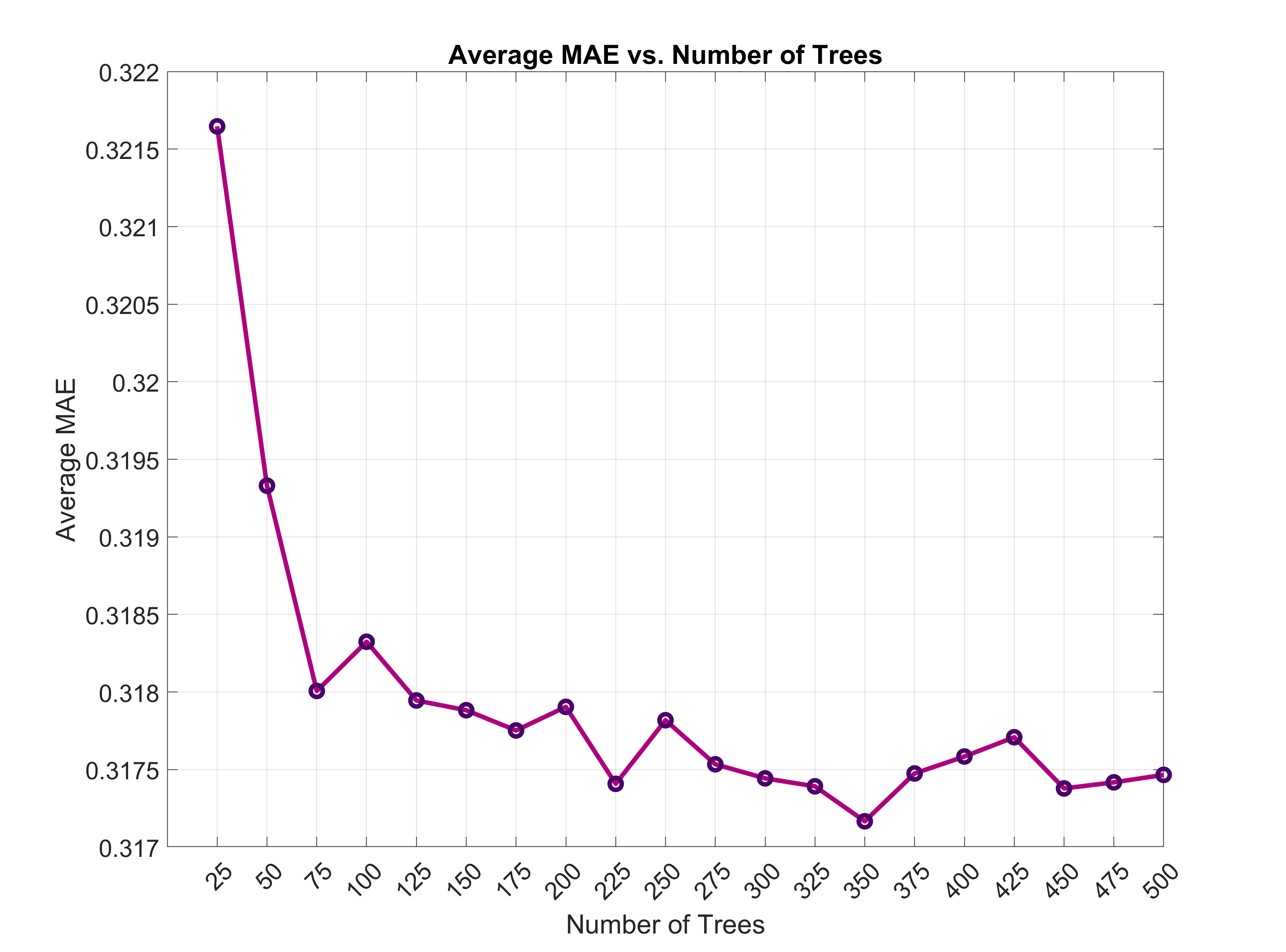

Supplement: S6 Fig — (TIF) [file pcbi.1013839.s008.tif]

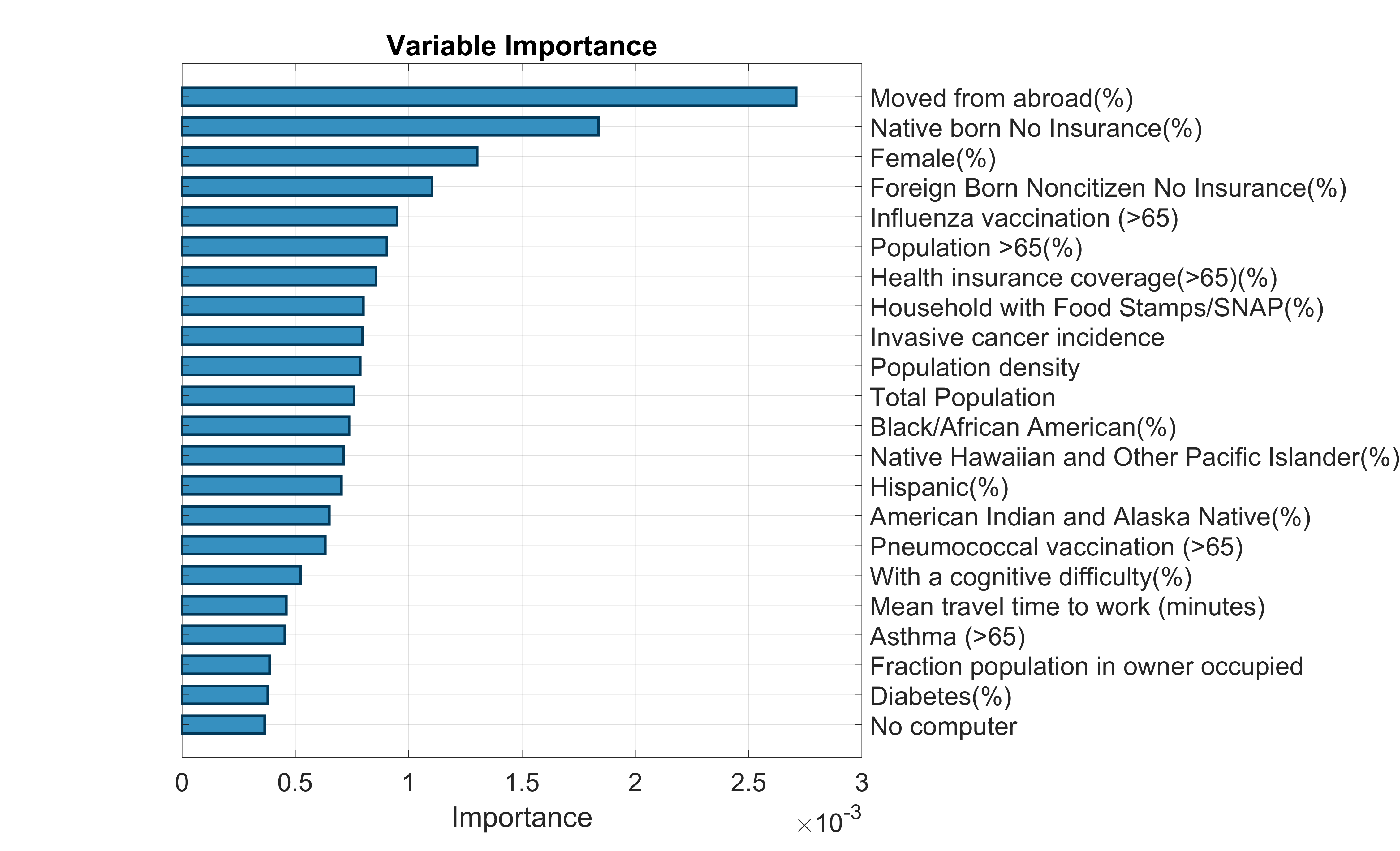

Supplement: S7 Fig — (TIF) [file pcbi.1013839.s009.tif]

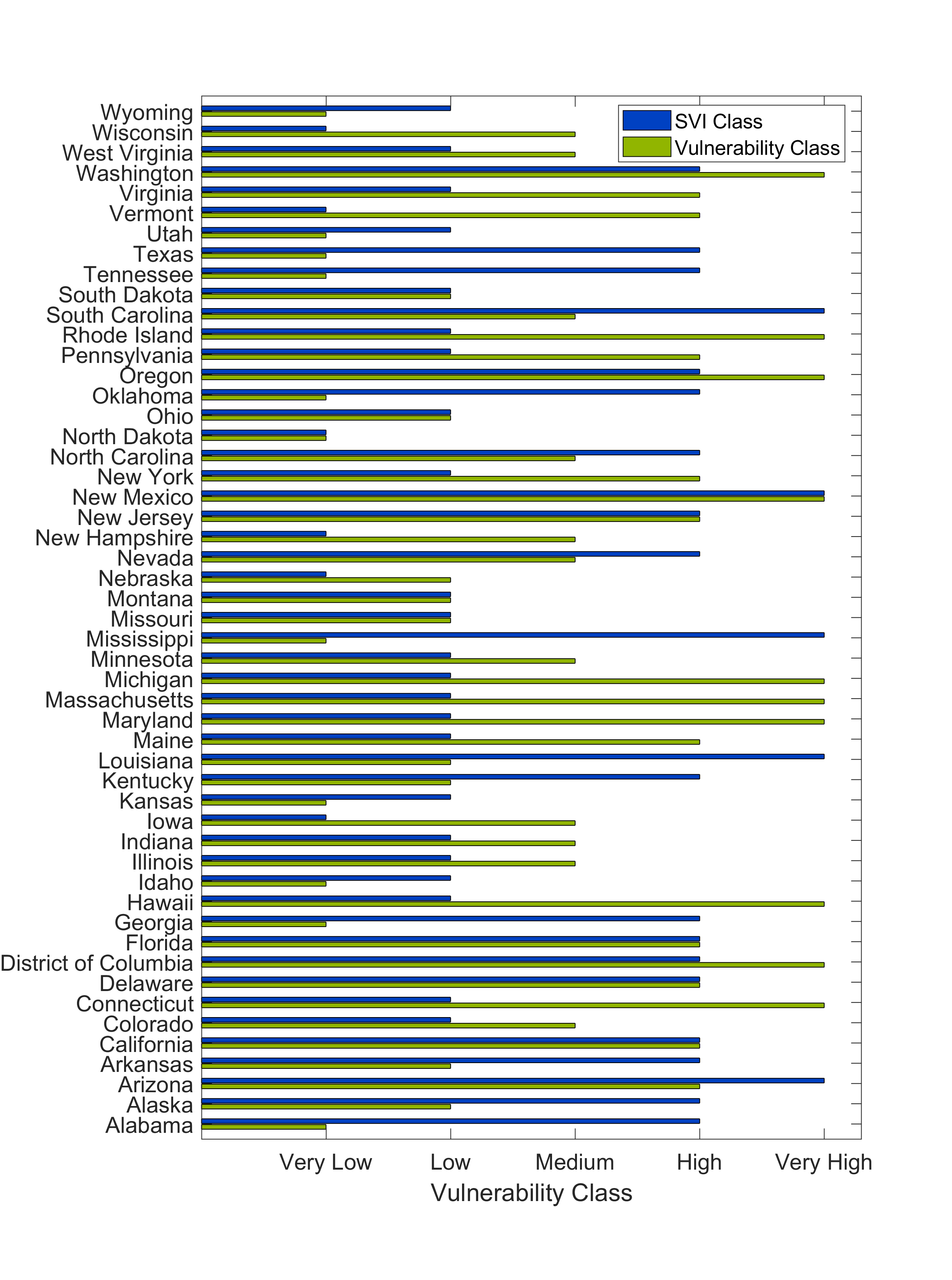

Supplement: S8 Fig — (TIF) [file pcbi.1013839.s010.tif]
