# Supplementary material for: Spatial variation in socio-economic vulnerability to Influenza-like Infection for the US population
Source: PLoS Comput Biol. 2026 Jan 28;22(1):e1013839. doi: 10.1371/journal.pcbi.1013839 (PMC12919934; doi:10.1371/journal.pcbi.1013839)
Supplement: Attachment — Submitted filename: Response to Reviewers.pdf [file pcbi.1013839.s011.pdf]

We sincerely thank the Editor and the reviewers for their valuable feedback and constructive suggestions. We have addressed each comment to the best of our knowledge. In the following document, we provide a point-by-point response to every comment. Our direct responses are presented in **blue text**, and corresponding changes made in the revised manuscript are in **blue and italics**.

Additional Editor Comments:

I agree with the reviewers that this is an interesting study that is likely of wide interest. As the authors note, we typically struggle to integrate measures of social vulnerability because they vary significantly over finer geographic scales than those associated with most of our infectious disease data. The reviewers provide a number of thoughtful comments that I strongly suggest the authors pay careful attention to during their revision. Looking across the reviewer comments, and considering my own evaluation, there is consensus that more work demonstrating the applicability of the aggregate social vulnerability measures would strengthen the manuscript considerably.

I would encourage the authors to consider directly comparing their measure to the CDC's Social Vulnerability Index (accomplishing this may require aggregating that measure appropriately).

We thank the editor for summarizing the paper as well as the reviewers' comments and providing their suggestions and feedback.

The CDC Social Vulnerability Index SVI is a static, general-purpose index designed to measure a community's vulnerability to a wide range of hazards, including natural disasters and public health emergencies. It's based on a fixed set of 16 census variables that are grouped into four distinct themes. This methodology calculates a percentile rank (from 0 to 1) for each of these four themes and an overall score for every U.S. census tract. It provides a consistent baseline for identifying communities that may need extra support, but it does not account for the unique epidemiological and socio-economic factors that drive the spread and severity of a specific infectious disease (CDC, 2022).

On the other hand, our approach offers a more precise and accurate assessment of socio-economic vulnerability to influenza-like infections compared to the CDC's SVI. While the SVI is a well-established and valuable tool for broad-spectrum disaster preparedness, its generic nature limits its effectiveness for a specific, rapidly spreading threat like influenza. Our model leverages machine learning to overcome these limitations. It is a dynamic, threat-specific approach which is more precise and accurate for the specific context of influenza. Unlike the SVI, machine learning

algorithms can learn complex, non-linear relationships between socioeconomic factors and vulnerability, and can be trained on specific outcomes influenza-like illness rates.

We have incorporated a brief description of the CDC SVI and a detailed comparison with our proposed model, which can be found in lines 100 - 113 of the manuscript. This section aims to highlight the core advantages of our novel approach. While existing methods use a common framework for all disasters, our model's specificity for influenza allows for a more precise and accurate assessment of vulnerability, representing a significant advancement over these more generic approaches. To illustrate the advantage of our approach, we compare our computed state-wise vulnerability values directly against the standard CDC SVI (Figure S8). This comparison demonstrates how incorporating machine-learning-derived, tailored indicator weighting provides a different vulnerability compared to a generic social vulnerability measure in most cases.

"CDC provides a very commonly used social vulnerability index (SVI) to natural disasters (CDC, 2022)*. It quantifies the degree to which a social condition may affect a region's ability to prevent loss in a disaster. Based on 16 social indictors (4 themes), CDC ranks each census tract and sums up the ranks to get the final rank of each location. Though this index provides a basic understanding of social vulnerability of a region, it has a major drawback. This index is generalized and treats responses to all natural disasters alike. Further, it considers individual values of each indicator to rank the vulnerability, assuming the same weight. However, in the case of a real disaster, different parameters play different roles based on the type of disaster. For example, during a coastal hurricane, home ownership and age of housing stock become primary drivers of total loss. However, in case of non-structural event like a pandemic, factors like access to healthcare and number of people with health insurance may drive the total loss. To overcome these drawbacks, here we propose a novel approach to use socio-economic indicators in a machine learning based approach to compute socio-economic vulnerability specific to Influenza like illness. Unlike the SVI, machine learning algorithms can learn complex, non-linear relationships between socioeconomic factors and vulnerability, and can be trained on specific outcomes influenza-like illness rates."*

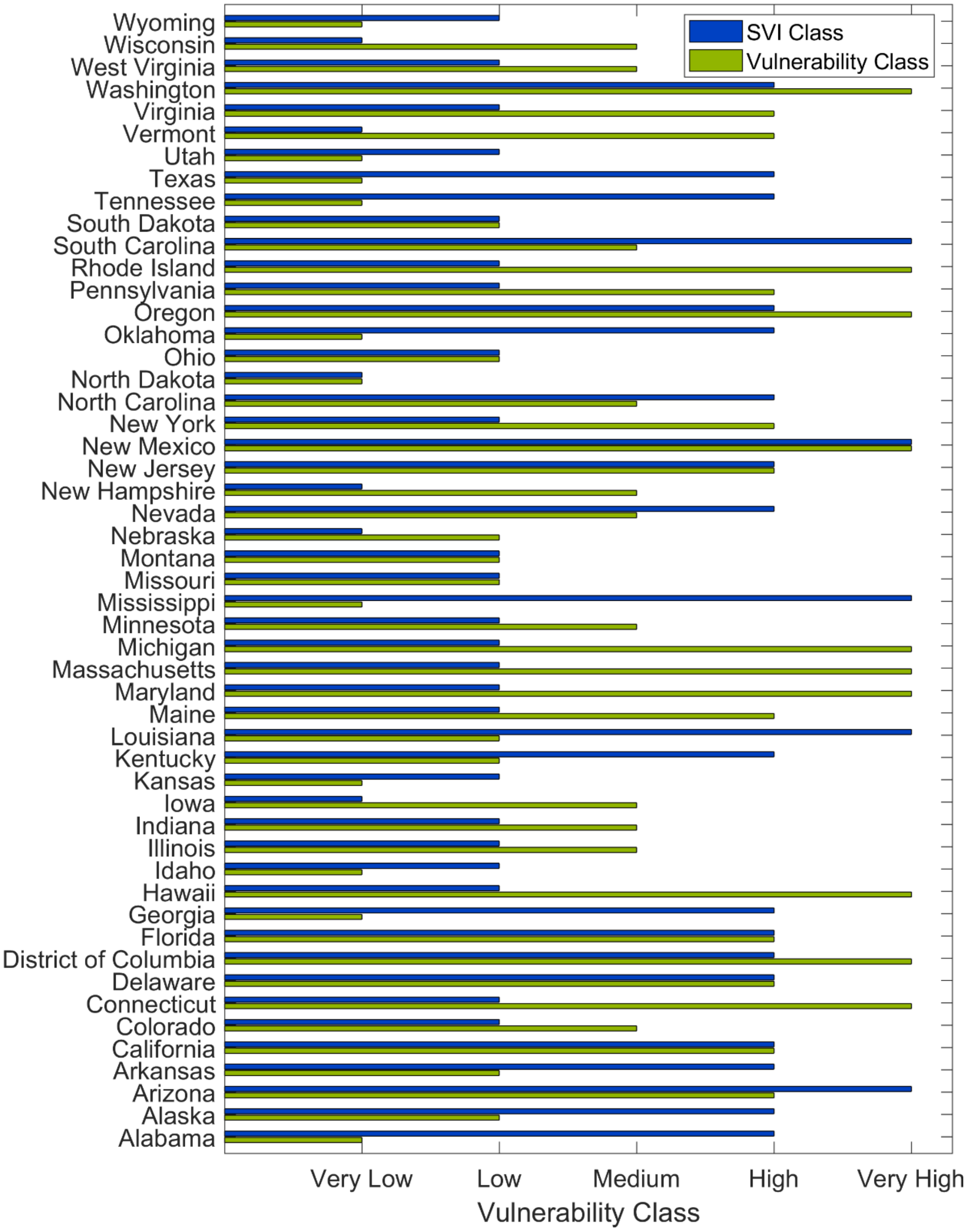

Figure S8: Comparison of Vulnerability and CDC SVI Classes by State

I agree with R2's comments that showing some pairwise relationships between components of the new aggregate measure and disease would aid with interpretation.

- Thank you for your suggestion regarding the need to analyse the individual relationships. To enhance the interpretability and aid in understanding the components of our aggregate vulnerability measure, we have included an examination of the pairwise relationships between each final predictor and the Influenza-like Illness (ILI) rate. The pairwise relationship is presented as a correlation matrix in Supplementary Figure S2 and is also discussed in the manuscript line 443-447.

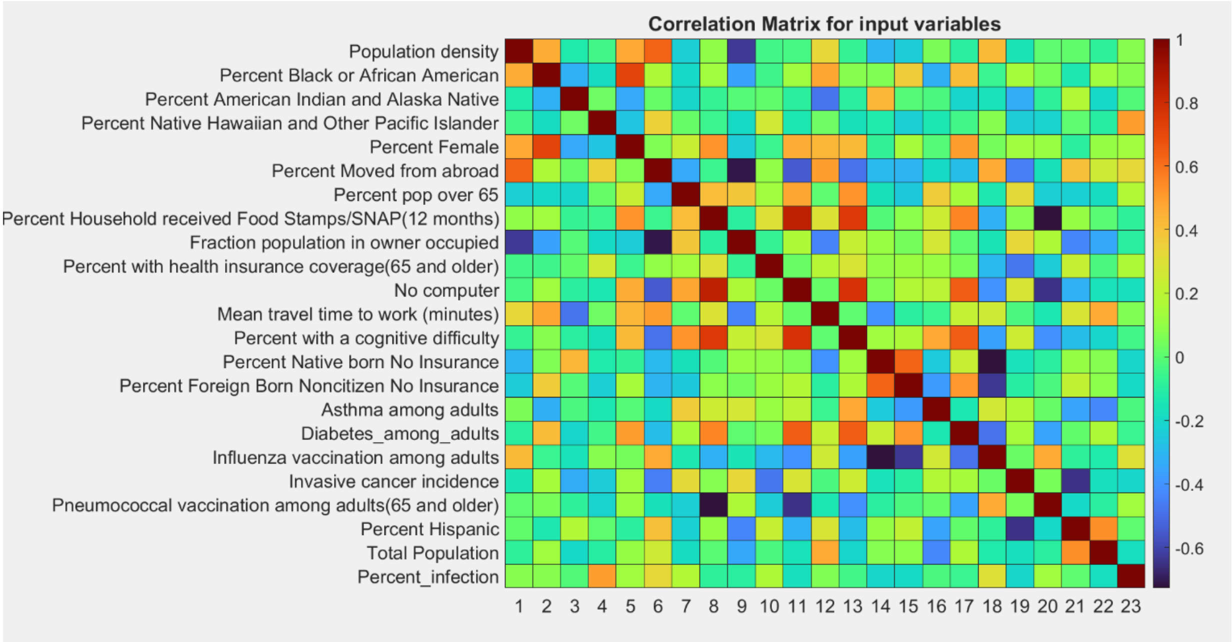

Figure S2. Pairwise Correlation Matrix for all 23 variables considered (22 independent input variables and 1 dependent variable (percent ILI infection)) with colour intensity indicating the strength and direction of the relationship.

"To understand the relationship between these indicators as well as the dependent variable (percent ILI infection) we also examined the pair-wise correlation matrix (Supplementary Figure S2). These reveal direct linear relationships between the independent and dependent variables providing immediate interpretation for the final aggregate vulnerability index."

More importantly, I am interested in how sensitive the results are to level of aggregation. While I realize you cannot look at ILI data sub-state-level, you can do so with the social vulnerability data. What would the results look like if you ran a kind of bootstrap strap analysis where you randomly selected census tracts to create 50 "pseudo-states" and use the resulting random forest models as a kind of null distribution?

We appreciate this comment. We understand the reviewer wants us to generate 50 pseudo states using census tract level data for the same 39 variables. The idea is to generate states and corresponding variable values by combining the census tract level values. So now we have independent variable values for 50 new states but no ILI value (dependent variable), and we can see how the model assigns vulnerability values to each state just based on the feature importance generated from the RF model trained with the original state level data.

Another option is generating pseudo states using census tract level socio-economic variables and using the model developed using the real states to generate ILI values for these pseudo states. However, to validate the results we would need ILI values, which are not available. Downscaled census tract level ILI values would not provide the real distribution of infection values, which is very much dependent on the socio-economic condition.

Could you train the model on 80% of the census tracts in states and predict aspects of social vulnerability at the census tract level in the 20% of census tracts that were held out?

- Thank you for your question. We are unable to train our model on census tracts to predict social vulnerability because our model's output is specifically vulnerability to influenza-like infections. This vulnerability is dependent on influenza infection data, which is only available at the state level, as discussed in the responses to the previous comments.

  With the current model and data, we have done training with the model with 80% of the data and testing with the remaining 20%. The analysis was iterated 100 times, by selecting different 80% datasets every time and taking an average RMSE value to understand the performance of the model. The resulting averaged out-of-sample has a Root Mean Squared Error (RMSE) 0.418. The same is also included to the manuscript in line 512-519.

"The Random Forest Regression (RFR) model is employed to determine the relative contribution of each socio-economic indicator. To provide a robust, out-of-sample estimate of predictive performance and to mitigate bias from a single split, we employed a repeated random holdout validation. This involved running 100 iterations, each utilizing an 80%/20% split of the data for

training and testing, respectively. The resulting average out-of-sample performance has a Root Mean Squared Error (RMSE) 0.418. However, the primary objective remains the stable identification and weighting of key vulnerability factors. The model successfully achieved this by yielding stable, non-zero feature importance scores for the final set of non-collinear predictors."

In terms of variable reduction, I would recommend comparing your method to commonly used dimensionality reduction approaches like principal component analysis.

- We acknowledge that Principal Component Analysis (PCA) is a powerful dimensionality reduction technique. PCA works by creating new, uncorrelated variables called principal components (PCs), which are linear combinations of the original variables. While this is effective for reducing dimensionality, the resulting PCs are often not directly interpretable as specific socio-economic indicators. Our primary goal is to not only predict vulnerability but also to interpret the relative importance of individual socio-economic factors. By using VIF to identify and remove highly correlated variables, we retain the original indicators, allowing us to understand their individual contributions to influenza vulnerability. This approach aligns with our goal of providing actionable insights for public health officials.

To this last point, it's not obvious to me why you need to perform dimension reduction when using a regularized approach like random forest (I can think of plausible reasons, but it would be useful to explain directly).

- Random Forest (RF) models are generally robust to multicollinearity and in general don't strictly require a preprocessing step like the Variance Inflation Factor (VIF). However, in this case, the presence of highly correlated variables can still lead to unstable and less interpretable variable importance scores. For instance, if two variables are highly correlated, the RF model might randomly split on either one, making it difficult to determine the true contribution of each. Our primary goal was to select a robust set of socio-economic indicators that are highly predictive of influenza vulnerability. To achieve this, we chose to use VIF as a preprocessing step, to filter out

highly colinear variables. (Hu et al., 2021; Strobl et al., 2008). We have added the same justification for implementing VIF in the revised manuscript as well (line 432 - 438).

"While Random Forest (RF) models are inherently robust to multicollinearity and do not strictly require pre-processing like the Variance Inflation Factor (VIF) for prediction, the presence of highly correlated predictor variables can destabilize the interpretation of feature importance (Hu et al., 2021; Strobl et al., 2008). Given that socio-economic vulnerability indicators often exhibit strong, internal collinearity, we aimed to select a feature set that offers a clearer, more independent understanding of contribution to Influenza-like Illness (ILI) vulnerability. To filter out highly collinear variables and ensure the statistical independence of our final predictor set, we incorporated VIF as a pre-processing step."

I did see that the authors referenced some supplemental material, but I was unable to access any supplement. Lastly, the authors state that data is publicly available. While it's true that data are publicly available, the intent behind the journal's policies are to facilitate reproducibility and allow others to effectively build from your work. I would strongly encourage the authors to make their data and analysis scripts publicly available via Github and archived with a DOI for some service like figshare or zenodo.

- We have uploaded the underlying data in the Mendeley Data repository accessible at the following reference: Tripathy, Shrabani (2025), "Underlying Data- ILI Vulnerability Analysis for USA", Mendeley Data, V1, doi: 10.17632/v4b2w26jbc.1

Reviewers' comments:
Reviewer's Responses to Questions

Comments to the Authors: Please note here if the review is uploaded as an attachment.

Reviewer #1: The manuscript presents a novel machine-learning-driven framework to assess and map state-level socio-economic vulnerability to Influenza-Like Illness (ILI) in the United States. By integrating 39 socio-economic and health indicators from U.S. Census and CDC data, the authors develop a vulnerability index using Random Forest Regression (RFR) to quantify regional disparities in ILI susceptibility. The study's focus on socio-economic determinants of health (SDOH) and its application of a data-driven approach to public health vulnerability assessment is a significant contribution to the field. The use of spatial mapping to visualize vulnerability enhances the practical utility of the findings for policymakers. However, several areas require clarification or improvement to strengthen the manuscript's methodological rigor, interpretative caution, and broader applicability. The use of Random Forest Regression to weight socio-economic and health indicators for a vulnerability index is a robust and replicable approach. The methodology leverages the non-linear modeling capabilities of RFR, which is well-suited for capturing complex interactions among SDOH. The

inclusion of 39 diverse indicators across seven dimensions (Income and Poverty, Population and People, Race and Ethnicity, Employment, Housing, Health, and Education) provides a holistic view of vulnerability, grounded in established SDOH literature. The manuscript occasionally employs language that implies causal relationships.

- We thank the reviewer for summarizing our work rather beautifully. We appreciate the encouraging words.

Random Forest models assess associations, not causation, and such statements risk overinterpretation. The authors should revise these claims to emphasize correlations and clarify the limitations of RFR in establishing causality.

- Thank you for this insightful comment. We completely agree that a Random Forest model, or any correlational model, can only assess associations and not establish causation. In the revised manuscript we have incorporated the directionality factor in our vulnerability quantification along with the weights for each variable. Based on partial dependence analysis, we have assigned directionality (d_k) to each variable. The directionality is computed based on the slope (positive or negative) and are quantified as +1 and -1. To incorporate the same in the vulnerability quantification we have modified the Equation (3) from the manuscript.
  Modified equation (3):

$$V_i = \sum_k w_k \times x'_{ik} \times d_k \text{ -------- (3)}$$

Where, w_k = Weight assigned to each vulnerability indicators (k)

V_i = Vulnerability Index at each state (i)

x'_{ik} = Value of variable k for state i

d_k = Directionality of variable k

d_k= 1 for positive slope
- 1 for negative slope

- The following table provides the directionality of each variable. We have also added the same to the supplementary information document as table S2.

Variable	Direction<br>(+1: Positive and -1: Negative)
Population density	+1: Positive
Percent Black or African American	+1: Positive
Percent American Indian and Alaska Native	+1: Positive
Percent Native Hawaiian and Other Pacific Islander	-1: Negative
Percent Female	+1: Positive
Percent Moved from abroad	+1: Positive
Percent pop (> 65)	+1: Positive

Percent Household received Food Stamps/SNAP (12 months)	+1: Positive
Fraction population in owner occupied	-1: Negative
Percent with health insurance coverage (> 65)	+1: Positive
No computer	+1: Positive
Mean travel time to work (minutes)	-1: Negative
Percent with a cognitive difficulty	+1: Positive
Percent Native born No Insurance	-1: Negative
Percent Foreign Born Noncitizen No Insurance	-1: Negative
Asthma among adults	-1: Negative
Diabetes among adults	-1: Negative
Influenza vaccination among adults	+1: Positive
Invasive cancer incidence	-1: Negative
Pneumococcal vaccination (>65)	+1: Positive
Percent Hispanic	+1: Positive
Total Population	-1: Negative

The same is also updated in the manuscript in Lines 544-551.

While the manuscript mentions the use of K-fold cross-validation and the selection of 200 decision trees based on minimized RMSE (Page 21, Line 373), it does not report specific model performance metrics (e.g., RMSE, R^2, or Mean Absolute Error) for the final RFR model.

- We thank the reviewer for this valuable comment. We have now clarified and expanded the description of the Random Forest Regression (RFR) model evaluation.
  The manuscript previously stated that 200 trees were used, which was a typographical error. The optimal model was trained using 450 decision trees, as determined through 10-fold cross-validation. The model's performance was evaluated in terms of the root mean square (RMSE) and mean absolute error (MAE) across different numbers of trees, and both Supplementary Figure S5 S6 now show that the RMSE stabilized around 450 trees, indicating convergence and optimal model complexity. MAE value for 450 is also in 2nd smallest. We have added the same to the manuscript as well in Line 464-468:

"The optimal number of Random Forest decision trees was determined using repeated 10-fold cross-validation across 200 simulation runs. The optimal number of trees was found to be 450, based on the stabilization of the averaged out-of-sample metrics: RMSE=0.3886 and

MAE=0.3174. The stability analysis for the number of trees, ranging from 25 to 500 (in increments of 25), is detailed in Supplementary Figures S5 and S6."

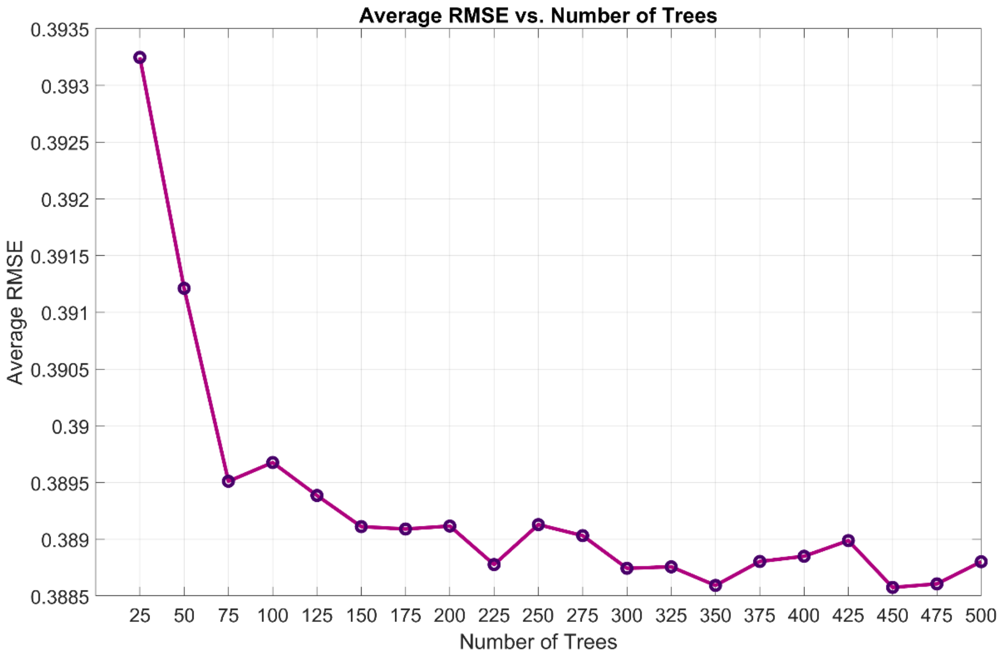

Figure S5: The root means square (RMSE) value corresponding to different number of decision trees selected for the RFR model.

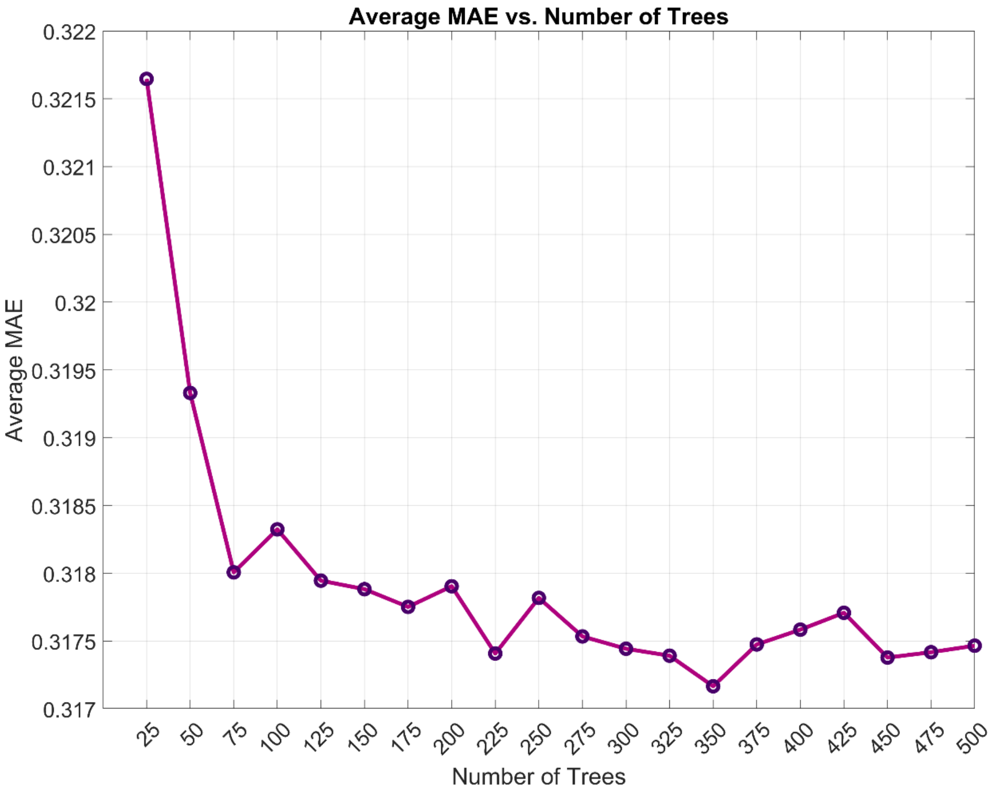

Figure S6: The mean absolute error (MAE) value corresponding to different number of decision trees selected for the RFR model.

Including a dedicated model validation section with quantitative metrics (e.g., test-set predictions, cross-validation scores, or bootstrapping results) would strengthen confidence in the model's predictive accuracy and generalizability.

- Thank you for the feedback regarding the need for a dedicated model validation section. We agree that rigorous quantitative metrics are essential for establishing confidence in the model's robustness and generalizability.

  To address this, we have implemented a repeated random holdout validation (100 × 80/20 splits). This robust, large-scale validation provides a stable estimate of the model's out-of-sample performance, ensuring the metrics are not based on a single, favorable data split. The final out-of-sample performance (100 x 80/20 Splits) is: RMSE: 0.418

  The same is also added to the revised manuscript in lines 512-519:

"The Random Forest Regression (RFR) model is employed to determine the relative contribution of each socio-economic indicator. To provide a robust, out-of-sample estimate of predictive performance and to mitigate bias from a single split, we employed a repeated random holdout validation. This involved running 100 iterations, each utilizing an 80%/20% split of the data for training and testing, respectively. The resulting average out-of-sample performance has a Root Mean Squared Error (RMSE) 0.418. However, the primary objective remains the stable identification and weighting of key vulnerability factors. The model successfully achieved this by yielding stable, non-zero feature importance scores for the final set of non-collinear predictors."

The rationale for selecting the initial 39 indicators and reducing them to 22 using Variance Inflation Factor (VIF) thresholding is insufficiently detailed (Page 20, Lines 319–349). The manuscript does not discuss the implications of excluding specific variables or justify the VIF threshold of 10. A more transparent explanation, supported by references or sensitivity analyses, would enhance the credibility of the variable selection process.

- Thank you for your valuable feedback regarding our variable selection process.

  Our primary goal was to select a robust set of socio-economic indicators that are highly predictive of influenza vulnerability based on past literature. While these indicators have strong association with the ILI, they may be highly colinear in nature, which needs to be minimized. Although Random Forest models are known for their ability to handle multicollinearity by randomly selecting feature subsets at each split, the presence of

highly correlated features can still impact the stability and interpretability of variable importance scores. Therefore, we chose to use Variance Inflation Factor (VIF) as a preprocessing step to ensure a more stable and interpretable RFR model (Hu et al., 2021; Strobl et al., 2008).

The selection of a VIF threshold of 10 is a commonly accepted practice in statistical modeling for identifying high multicollinearity (Gu et al., 2023; Li & Demir, 2023). Variables with VIF scores above this threshold are often considered redundant or highly correlated with other indicators. Removing these variables did not reduce the model's predictive power but significantly improved the clarity of the variable importance rankings, allowing us to focus on the most impactful and non-redundant socio-economic factors. We tried to find the best combination of variables so that the VIF value remains below 10. For full transparency, we've included a table in the supplementary material listing all 39 initial variables. Their VIF values are further illustrated in figures in the supplementary document (Figures S3 and S4) which provides a VIF value associated with each variable. The same is also included in the revised manuscript line 465-476:

"While Random Forest (RF) models are inherently robust to multicollinearity and do not strictly require pre-processing like the Variance Inflation Factor (VIF) for prediction, the presence of highly correlated predictor variables can destabilize the interpretation of feature importance (Hu et al., 2021; Strobl et al., 2008). Given that socio-economic vulnerability indicators often exhibit strong, internal collinearity, we aimed to select a feature set that offers a clearer, more independent understanding of contribution to Influenza-like Illness (ILI) vulnerability. To filter out highly collinear variables and ensure the statistical independence of our final predictor set, we incorporated VIF as a pre-processing step. We analyzed the VIF values for all 39 indicators in Supplementary Figure S3. To ensure statistical independence of the final predictor set, a VIF threshold of 10 was employed, a widely accepted practice in statistical modeling for identifying high multicollinearity (Gu et al., 2023; Li & Demir, 2023). *After systematically removing variables exceeding this threshold, the resulting feature set was reduced from 39 to 22 variables. The VIF values for these remaining non-collinear variables are presented in Supplementary Figure S4."*

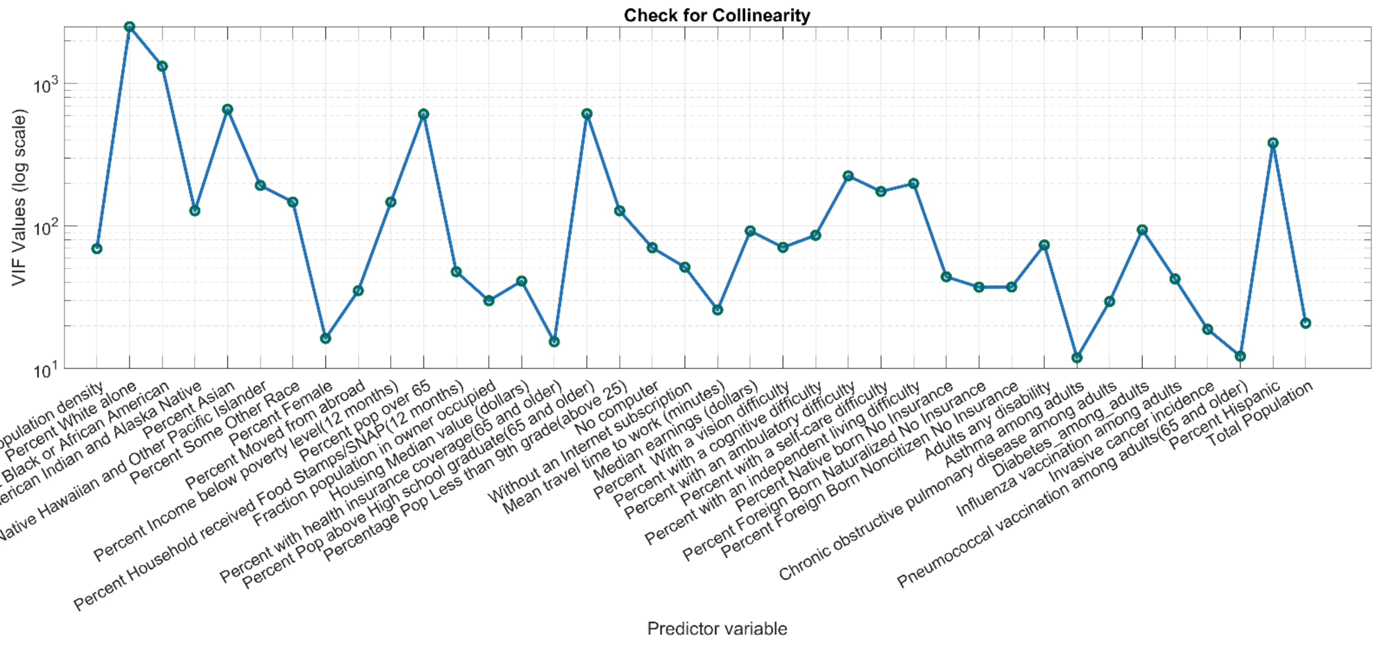

Figure S3: VIF values for all 39 indicators

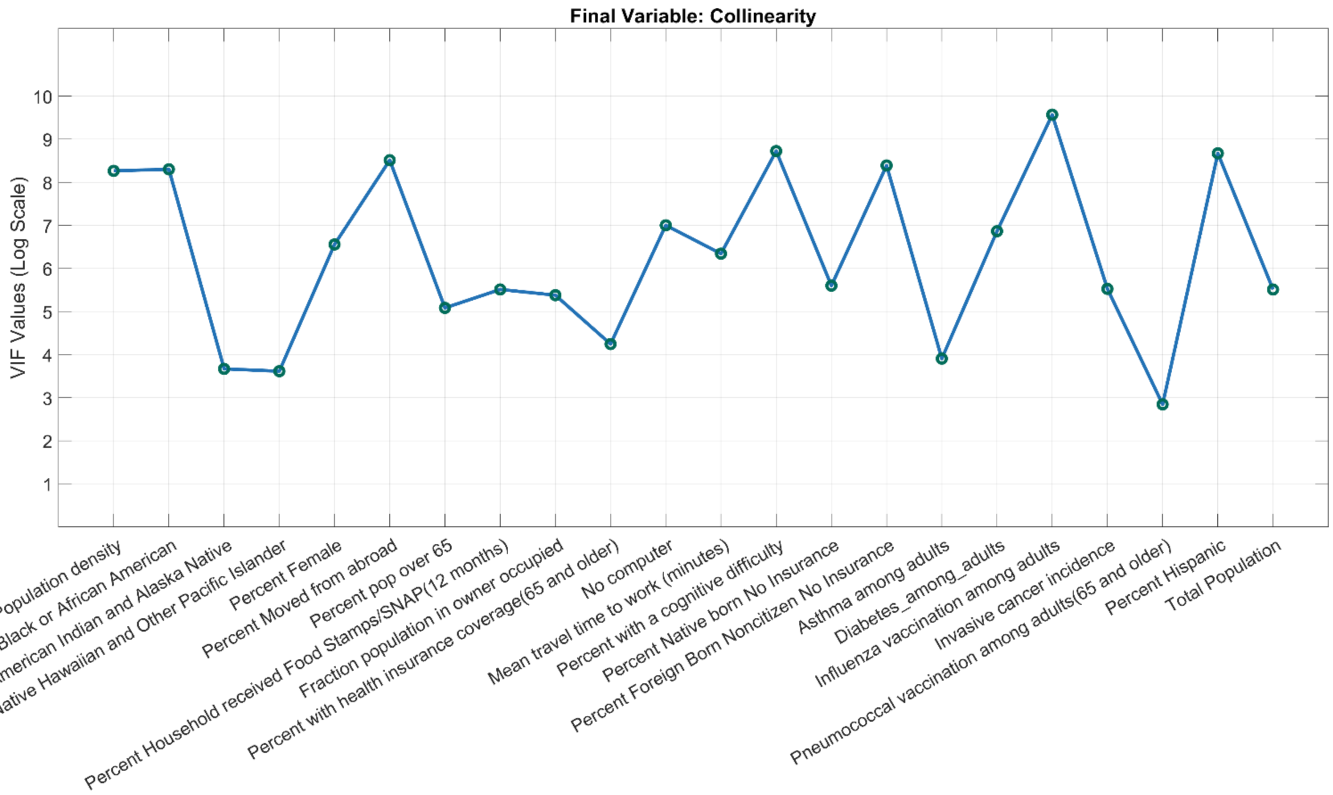

Figure S4: VIF values for the final 22 indicators. All the variables have values less than 10.

Additionally, Supplementary Table 1, referenced for justification, is not provided in the document, limiting the ability to evaluate the scientific rigor of the selection.

- We apologize for the technical issue that might have prevented the supplementary file from being included in the original submission. We have corrected this and can confirm that the supplementary document, including Table S1, is now provided with the revised manuscript. We thank you for pointing out this omission.

The study's geographic framing would benefit from testing for spatial autocorrelation in model residuals, using metrics such as Moran's I or Local Indicators of Spatial Association (LISA). This analysis would confirm whether the RFR model fully captures regional variations in ILI vulnerability or if unmeasured spatial processes (e.g., regional policy differences, healthcare infrastructure gaps, or cultural factors) influence the results. Significant spatial clustering in residuals could suggest the need for spatial modeling (e.g., Geographically Weighted Regression) or multilevel approaches in future work.

- We appreciate the reviewer's insightful suggestion to test for spatial autocorrelation in the model residuals. To evaluate whether unmodeled spatial processes might influence our results, we computed Global Moran's I and Local Indicators of Spatial Association (LISA) using the centroids of the U.S. states.

  The results were as follows:

  Number of States (N): 52
  Moran's I Statistic: -0.0435
  Expected Value E[I]: -0.0196
  Z-Score: -2.87
  P-Value (Two-Tailed): 0.0041
  Result: Significant NEGATIVE spatial autocorrelation (dispersion)

  As shown in Figure S1, the residuals exhibit a weak but statistically significant negative spatial autocorrelation, indicating spatial dispersion rather than clustering. This suggests that neighboring states tend to have dissimilar residual values, implying that the RFR model effectively captures most of the spatially structured variability in ILI vulnerability.

  Given this dispersion pattern, there is no indication of unmodeled spatial clustering that would necessitate spatial regression or geographically weighted modeling at this scale. Nevertheless, we acknowledge that future extensions of this work could benefit from exploring spatial or multilevel modeling frameworks, especially with finer-resolution data (e.g., county-level or census-tract level).

The same is added to the manuscript as well (Lines 185-190).

"To evaluate whether unmodeled spatial processes might influence our results, we computed Global Moran's I and Local Indicators of Spatial Association (LISA) using the centroids of the U.S. states. As presented in supplementary Figure S1, the residuals exhibit a weak but statistically significant negative spatial autocorrelation (Moran's I = –0.0435, p = 0.0041), indicating spatial dispersion rather than clustering. This suggests that neighboring states tend to have dissimilar residual values, implying that the RFR model effectively captures most of the spatially structured variability in ILI vulnerability."

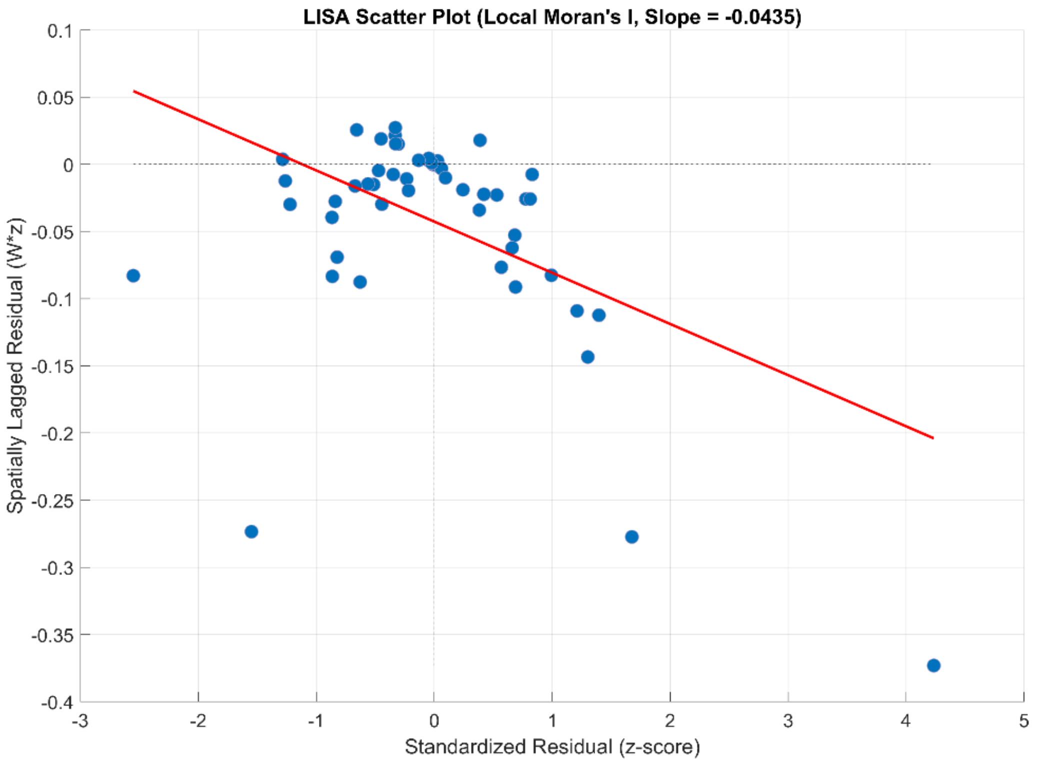

Figure S1: LISA (Local Moran's I) scatter plot of RFR model residuals across 52 U.S. states. The negative slope (Moran's I = –0.0435, p = 0.0041) indicates significant spatial dispersion, suggesting that neighbouring states tend to exhibit dissimilar residual values, with no evidence of spatial clustering.

Aggregating SDOH at the state level may obscure significant intra-state variations, particularly in large or socio-economically diverse states like California, Texas, or New York (Page 9, Line 123). While the authors acknowledge the potential for finer-scale analyses (e.g., county or ZIP code level), they do not discuss how state-level aggregation might mask local disparities, which is a critical limitation in SDOH research. For example, socio-economic factors like poverty or healthcare access often operate at the community or county level, and their impact may be diluted or misrepresented at the state level. The manuscript should include a discussion of this limitation and consider evaluating the relevance of certain indicators (e.g., population density, healthcare access) at the state level versus finer scales.

We thank the reviewer for highlighting the critical limitation concerning state-level aggregation of Social Determinants of Health (SDOH) and the potential for obscuring significant intra-state variations in large or diverse states (e.g., California, Texas). We have included a detailed discussion of this constraint in the revised manuscript (Lines 368-377).

Despite this data constraint, the methodology presented here, a data-driven approach using Random Forest for the construction of an interpretive index, is universal and scale-independent. The relative feature importance assigned to the key socio-economic indicators, derived from the state-level signal, reflects the underlying mechanism of vulnerability. We hypothesize that this relative ranking of factors should generally hold true even when applied to finer spatial scales. Moreover, the methodology can be directly applied to any spatial scale (county, census tract, etc.) once the corresponding sub-state ILI rate data becomes available. While the current state-level vulnerability map is limited by data availability, the underlying feature-weighted index methodology remains a generalizable framework for assessing relative susceptibility based on social and economic characteristics.

"This study demonstrates the variability of vulnerability to Influenza-like Illness (ILI) across different states. However, it is essential to acknowledge that this state-level aggregation is coarse and may not fully represent the actual socio-economic heterogeneity that exists within states. Socio-economic characteristics, and thus vulnerability, often vary significantly on a finer scale, such as the community or neighborhood level. While the resulting state-level vulnerability map is limited by the unavailability of finer-scale ILI data, the underlying feature-weighted index methodology remains a universal and generalizable framework for assessing relative susceptibility. Furthermore, the feature importance assigned to the key socio-economic indicators is hypothesized to reflect the fundamental mechanisms of vulnerability and can be extended to understand sub-state level variation even if the dependent variable data for that scale is currently absent."

The manuscript makes a compelling contribution to public health by introducing a machine-learning-driven framework for assessing socio-economic vulnerability to ILI. The use of RFR

and spatial mapping is innovative and has significant potential for informing targeted interventions. However, addressing the concerns regarding causal language, model validation, indicator selection, spatial autocorrelation, and state-level aggregation will significantly strengthen the manuscript's scientific rigor and impact. With these revisions, the study could serve as a valuable resource for public health researchers and policymakers aiming to mitigate infectious disease disparities.

- We are grateful for the encouraging remarks.

Reviewer #2: Overall, the authors attempt to answer an important questions through a novel framework. My biggest concern is around the assumption of direction, more or less vulnerable, based on variable importance outputted from random forest regression. Random forests do not allow for interpretation of relationship, i.e. although a variable is important to the outcome it is not clear if it has a positive or negative relationship. My suggestion would be to add more descriptions or supplemental tables/images showing the relationship between each indicator used to create the social vulnerability index and the main outcome of the regression model, I think ILI, by doing univariate analysis or even multivariate since multicollinearity is taken care of in variable selection. This will assure the reader that the assumed "more vulnerable" relationship is valid.

- We thank the reviewer for the valuable suggestion. We have incorporated directionality in the vulnerability quantification. Based on partial dependence analysis, we have assigned directionality to each variable. The directionality is computed based on the slope and assigned as +1 or -1. To incorporate the same in the vulnerability quantification we have modified the Equation (3) from the manuscript.
  Modified equation (3):

$$V_i = \sum_k w_k \times x_{ik} \times d_k \text{ -------- (3)}$$

  Where, w_k = Weight assigned to each vulnerability indicator (k)

  V_i = Vulnerability Index at each state (i)

  x_{ik} = Value of variable k for state i

  d_k = Directionality of variable k

  d_k= 1 for positive slope
  - 1 for negative slope

- The following table provides the directionality of each variable. We have also added the same to the Supplementary Information in Table S2.

Variable	Direction (+1: Positive and -1: Negative)
Population density	+1: Positive
Percent Black or African American	+1: Positive
Percent American Indian and Alaska Native	+1: Positive

Percent Native Hawaiian and Other Pacific Islander	-1: Negative
Percent Female	+1: Positive
Percent Moved from abroad	+1: Positive
Percent pop (> 65)	+1: Positive
Percent Household received Food Stamps/SNAP (12 months)	+1: Positive
Fraction population in owner occupied	-1: Negative
Percent with health insurance coverage (> 65)	+1: Positive
No computer	+1: Positive
Mean travel time to work (minutes)	-1: Negative
Percent with a cognitive difficulty	+1: Positive
Percent Native born No Insurance	-1: Negative
Percent Foreign Born Noncitizen No Insurance	-1: Negative
Asthma among adults	-1: Negative
Diabetes among adults	-1: Negative
Influenza vaccination among adults	+1: Positive
Invasive cancer incidence	-1: Negative
Pneumococcal vaccination (>65)	+1: Positive
Percent Hispanic	+1: Positive
Total Population	-1: Negative

The same is also updated in the manuscript in Lines 544-551.

Abstract

- "To address multicollinearity, VIF was applied." Do you mean "to assess" and "was calculated"?

- Thank you for the comment we have updated on the same in Line 22.

-I suggest adding the timeframe of the study into the abstract, what influenza season?

- Thank you for the comment we have updated on the same in Line 22.

Introduction

-I suggest adding an example to socio-economic structures on line 56 in the Introduction. A simple ",like ....." may be nice for the readers.

- We appreciate this suggestion; we have rewritten the section to provide a better understanding of the socio-economic structure. Added the same to lines 61 - 65:

"For example, some regions with high population density, limited healthcare access, or low income are more vulnerable to health crises than others with lower population density or higher average income. Vulnerability assessment for health risk then allows the relative susceptibility of these different regions to be quantitatively understood and compared."

- I suggest defining more clearly what “regions” your study looks at in the introduction’s 3rd paragraph, i.e. state. Region is quite vague to be the only term used in that paragraph.

- We thank the reviewer for pointing out the potential ambiguity. We have updated the terminology in the updated manuscript.

-I suggest moving the 5th paragraph in the introduction to be added on the 2nd paragraph since it nicely gives the introduction on ILI and vulnerability all at once.

- We have updated the same in the revised manuscript.

-I suggest adding your timeframe into the last paragraph of the introduction, i.e. what time frame your study looks at in the US.

- Thank you for the suggestion. We have added the following line to clarify the year in lines 150-152:

“We use state-specific ILI data for the year 2022 (Figure 1), which corresponds to the period for which most of the socio-economic data is available.”

-I recommend either discussing the make-up of ATSDR’s social vulnerability index in the introduction or in the discussion for comparison. https://www.atsdr.cdc.gov/place-health/php/svi/index.html

- In the original manuscript we mentioned it briefly in the introduction. In the revised manuscript we have elaborated on this in the introduction (lines 100-113) to provide a more holistic understanding of the existing more popular indices and our proposed method.

“CDC provides a very commonly used social vulnerability index (SVI) to natural disasters (CDC, 2022). It quantifies the degree to which a social condition may affect a region’s ability to prevent loss in a disaster. Based on 16 social indictors (4 themes), CDC ranks each census tract and sums up the ranks to get the final rank of each location. Though this index provides a basic understanding of social vulnerability of a region, it has a major drawback. This index is generalized and treats responses to all natural disasters alike. Further, it considers individual values of each indicator to rank the vulnerability, assuming the same weight. However, in the case of a real disaster, different parameters play different roles based on the type of disaster. For example, during a coastal hurricane, home ownership and age of housing stock become

primary drivers of total loss. However, in case of non-structural event like a pandemic, factors like access to healthcare and number of people with health insurance may drive the total loss. To overcome these drawbacks, here we propose a novel approach to use socio-economic indicators in a machine learning based approach to compute socio-economic vulnerability specific to Influenza like illness. Unlike the SVI, machine learning algorithms can learn complex, non-linear relationships between socioeconomic factors and vulnerability, and can be trained on specific outcomes influenza-like illness rates."

Results
-I suggest adding what the regression model's main outcome was. I think it helps clarify for the reader to understand the weights.

- We appreciate the comment and have updated the same in the method section of the updated manuscript (Line 516)
  "The resulting average out-of-sample performance has a Root Mean Squared Error (RMSE) 0.418."

-It seems that figure 1 goes with the method description, which is after the results section. Please align images with current text placement.

- We appreciate the suggestion to align figure placement with the corresponding text. However, in the modified manuscript, we have discussed the 2022 ILI data in the introduction section and have mentioned the figure in line 151.

"We use state-specific ILI data for the year 2022 (Figure 1), which corresponds to the period for which most of the socio-economic data is available."

-Figure 3's legend needs more description about what the numbers are. Weights or proportions or feature importance? It is unclear.

- The legend is updated in the revised manuscript in lines 194-199:

*"Figure 3: **Indicators for vulnerable states**: This figure presents radar plots to compare the normalized values of the 22 indicators across three states very highly vulnerable to Influenza-like Illness (ILI): (a) District of Columbia (DC) (urban), (b) Michigan (MI) (mixed), and (c) New Mexico (NM) (rural). The value on each section represents the indicator's normalized score (0 to 1). The Purple segments indicate factors that increase vulnerability (positive directionality), and orange segments indicate factors that decrease vulnerability (negative directionality)."*

-On lines 209-211, you refer to figure 4 but also to weights. Are there weights in figure 4? It is

unclear if the normalized value of the indicator is the same as weights or different or what that relationship is.

- We understand the potential confusion regarding normalized value of the indicators and the weights. A detailed discussion can be found in the methodology section. Figure 4 displays normalized data, indicated by the size of the scatter plots and the indicators in the X-axis are arranged in the order of weights.

  Normalized Indicator Value (x_{ik}) is the input data for a specific indicator k in a specific state i, scaled between 0 and 1. The normalized value varies for every state, reflecting the state's unique socio-economic status. This is the value presented in Figure 4 as the size of the scatter plot. Whereas weight (w_k) is derived directly from the Feature Importance (FI_k) determined by the random forest model. It represents the relative importance of indicator k. The weight w_k is a constant value applied uniformly to all states in the final index calculation.

  We have revised the figure caption and the following discussion to indicate the same Lines 243-245.

*"Figure 4: **State-Level Indicator Trends and Vulnerability.** This scatter plot displays normalized socio-economic and health indicators (0 to 1) across U.S. states. The size of each data point (bubble) corresponds to the normalized value of the indicator(x_{ik}), while the color represents the state's vulnerability class. The x-axis presents the indicators in descending order of RFR-derived feature importance or weight (w_k), ranging from "Moved from abroad (%)" (highest importance) to "No computer" (lowest importance)."*

"The weight w_k is a constant value applied uniformly to all states in the final index calculation. This weight value(w_k) combined with the normalized value (x_{ik}) of the indicator gives the vulnerability index for each state (V_i) as given in Eq (3) in the methodology section."

-I suggest presenting a visual, table or supplement to explain the importance and weights for each indicator.

- This is a great suggestion which will improve the overall understanding of the paper. We have now included Supplementary Figure S7 in the supplementary material which provides the value of weights for each indicator. The same is also mentioned in the revised manuscript (lines 521-523).

"The FI values corresponding to each indicator are presented in Supplementary Figure S7. This score does not directly indicate the value of vulnerability."

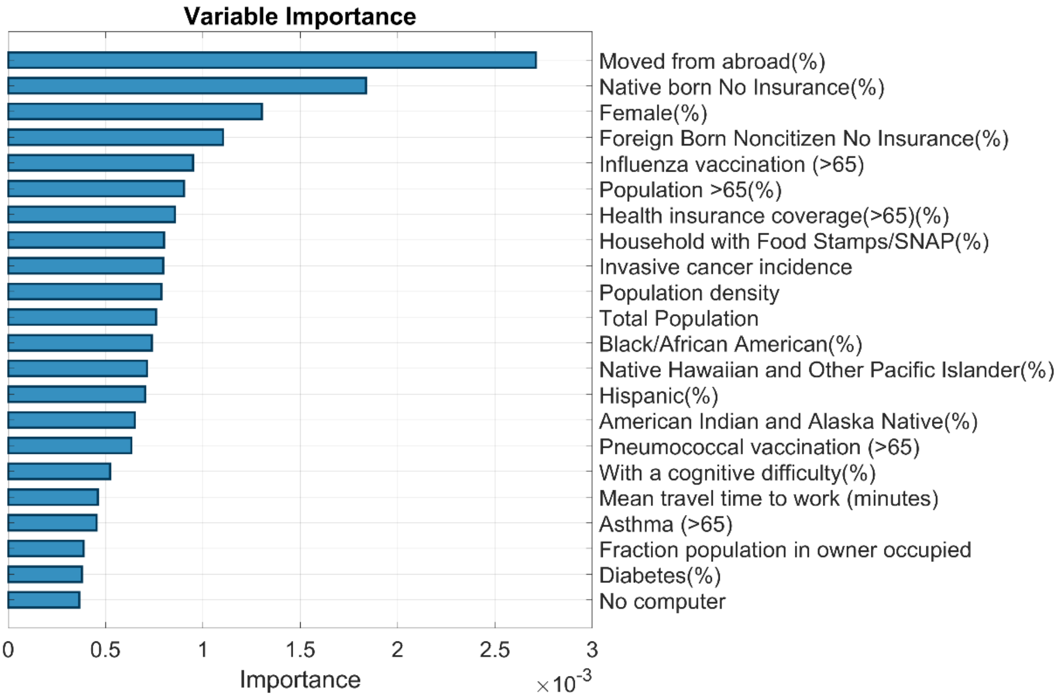

Figure S7: Final Set of Predictor Variables and their Importance for Influenza Vulnerability computed using random forest

Discussion
-Please add limitations.

- The limitations of the study were briefly discussed in the original manuscript at the end of the discussion section. In the revised manuscript we have highlighted the same in more detail in a dedicated paragraph in the discussion section. Line 368-381:

"This study demonstrates the variability of vulnerability to Influenza-like Illness (ILI) across different states. However, it is essential to acknowledge that this state-level aggregation is coarse and may not fully represent the actual socio-economic heterogeneity that exists within states. Socio-economic characteristics, and thus vulnerability, often vary significantly on a finer scale, such as the community or neighborhood level. While the resulting state-level vulnerability map is limited by the unavailability of finer-scale ILI data, the underlying feature-weighted index methodology remains a universal and generalizable framework for assessing relative susceptibility. Furthermore, the feature importance assigned to the key socio-economic indicators is hypothesized to reflect the fundamental mechanisms of vulnerability and can be extended to understand sub-state level variation even if the dependent variable data for that scale is currently absent. Also, the proposed socio-economic vulnerability quantification is inherently limited by the selection of indicators, since feature importance values are computed based on the socio-economic factors selected. From a methodological standpoint, the predictive performance of the Random Forest Regression (RFR) model is

constrained by the small sample size (N=50); incorporating a larger number of data points would be necessary to significantly improve its predictive accuracy."

-Since you did not have other respiratory indicators in the random forest, like weather or schooling data or mobility data, then you cannot say that this probes socio-economic indicators shape ILI more than any other indicators. This is because you only looked at socio-economic indicators. Therefore, you can only have relative importance noted between the indicators you investigated. For example, if you had put in humidity data and that was the most important variable then the message would be that humidity is more important than socio-economic. Just make sure all of the discussion and results are relative to only the indicators you investigated. It may be worth it to note this in the limitations.

- We agree with the reviewer that the conclusions regarding feature importance are relative to the set of indicators considered. Since the Random Forest model was intentionally constrained to analyse only socio-economic indicators derived from Census data, we cannot make claims about their importance compared to exogenous factors like weather, schooling, or mobility.

  Our study's objective was specifically to quantify socio-economic vulnerability. Therefore, we focused on using reliable social and economic parameters The analysis started with 39 variables (listed in Supplementary Table S1) including "Population 65 years and over- High school graduate or higher". Out of these, final 22 variables were selected solely based on their low multicollinearity (via VIF filtering).

  We have ensured that all discussion and interpretation of variable importance is framed as relative importance among the investigated socio-economic indicators. Furthermore, we have added this as a Limitation to the revised manuscript in Lines 377-379.

"Also, the proposed socio-economic vulnerability quantification is inherently limited by the selection of indicators. As all importance findings are strictly relative to the socio-economic factors investigated."

-Limitation to interpret the variables relationship to ILI from a random forest regression. For example, % female may have a string relationship with ILI in a positive way, i.e. the lower the proportion the higher the ILI. It should be clear that the limitation from random forest variable importance is not being able to infer a relationship direction.

- We agree with the reviewer on this. In the revised manuscript we have incorporated the directionality factor in our vulnerability quantification along with the weights for each variable. Based on partial dependence analysis, we have assigned directionality (d_k) to each variable. The directionality is computed based on the slope (positive or negative) and are quantified as +1 and -1. To incorporate the same in the vulnerability quantification we have modified the Equation (3) from the manuscript.
  Modified equation (3):

$$V_i = \sum_k w_k \times x_{ik} \times d_k \text{ -------- (3)}$$

Where, w_k = Weight assigned to each vulnerability indicators (k)

V_i = Vulnerability Index at each state (i)

x_{ik} = Value of variable k for state i

d_k = Directionality of variable k

d_k = 1 for positive slope
- 1 for negative slope

- The following table provides the directionality of each variable. We have also added the same to the supplementary information document as table S2.

Variable	Direction (+1: Positive and -1: Negative)
Population density	+1: Positive
Percent Black or African American	+1: Positive
Percent American Indian and Alaska Native	+1: Positive
Percent Native Hawaiian and Other Pacific Islander	-1: Negative
Percent Female	+1: Positive
Percent Moved from abroad	+1: Positive
Percent pop (> 65)	+1: Positive
Percent Household received Food Stamps/SNAP (12 months)	+1: Positive
Fraction population in owner occupied	-1: Negative
Percent with health insurance coverage (> 65)	+1: Positive
No computer	+1: Positive
Mean travel time to work (minutes)	-1: Negative
Percent with a cognitive difficulty	+1: Positive
Percent Native born No Insurance	-1: Negative
Percent Foreign Born Noncitizen No Insurance	-1: Negative
Asthma among adults	-1: Negative
Diabetes among adults	-1: Negative
Influenza vaccination among adults	+1: Positive
Invasive cancer incidence	-1: Negative
Pneumococcal vaccination (>65)	+1: Positive
Percent Hispanic	+1: Positive
Total Population	-1: Negative

The same is also updated in the manuscript in Lines 544-551.

Methodology
-Be very careful with interpreting variable importance as indicating vulnerability.

- We agree that direct, isolated interpretation of machine learning variable importance as a vulnerability can be misleading. We ensure throughout the manuscript not to interpret the feature importance to indicate vulnerability directly. The same is not indicated in the manuscript in Lines 522

"This score does not directly indicate the value of vulnerability."

-Do you use 1 peak week data for each state? So the data going into the model is only 50 observations? Why not use the entire time series? Please explain in the methods. Also, I would suggest using multiple seasons then.

- We used the maximum weekly Influenza-like Illness (ILI) infection rate for each state as the dependent variable for the Random Forest model. This approach results in a dataset of 50 observations (N=50), representing one data point per state. The peak infection rate best represents the maximum acute burden on a state's healthcare system and economy. This aligns with our goal of identifying socio-economic factors that amplify susceptibility during the most critical periods.
  The primary objective is not to model the dynamic, time-series progression of ILI (which would require incorporating dynamic variables), but to assess which foundational and static socio-economic factors make a state susceptible to ILI. Quantifying this seasonal or annual temporal variability is beyond the scope of the current study, which aims to provide a single, interpretive vulnerability map driven by socio-economic indicators.

  While extending the analysis to include multiple seasons or the entire time series is a valuable suggestion for future work, it would transition the analysis into a spatio-temporal modelling effort. We have added the same in lines 391-394 as a future scope of the study.

"While the current methodology successfully identifies static socio-economic drivers, future research should transition to a spatio-temporal modeling effort that incorporates the entire time series and multiple seasons to explicitly quantify the dynamic and temporal variability of ILI vulnerability."

Reviewer #3:

General Comments: The present study aims to quantify environmental health impacts and assess risk by examining the disproportionate burden of Influenza-Like Illness (ILI). While the manuscript has potential and offers novel contributions, it requires substantial revisions before it can be considered for publication.

Major Comments

The introduction is too general. It would be more effective if the authors focused on the burden of flu-like diseases. The magnitude and significance of the problem should also be clearly articulated in the introduction. The manuscript does not adequately address the rich body of existing literature on SVI and influenza interventions (e.g., vaccination). To strengthen the paper, key references should be incorporated. For instance, the following works highlight the magnitude and significance of the problem and may be added:

Tatar, M., Faraji, M. R., & Wilson, F. A. (2023). Social vulnerability and initial COVID-19 community spread in the US South: A machine learning approach. BMJ Health & Care Informatics, 30(1), e100703.

O'Sullivan, T., & Bourgoin, M. (2010). Vulnerability in an influenza pandemic: Looking beyond medical risk. Behaviour, 11(16).

Nayak, A., Islam, S. J., Mehta, A., Ko, Y. A., Patel, S. A., Goyal, A., ... & Quyyumi, A. A. (2020). Impact of social vulnerability on COVID-19 incidence and outcomes in the United States. MedRxiv, 2020-04.

Khazanchi, R., Beiter, E. R., Gondi, S., Beckman, A. L., Bilinski, A., & Ganguli, I. (2020). County-level association of social vulnerability with COVID-19 cases and deaths in the USA. Journal of General Internal Medicine, 35(9), 2784–2787.

- We thank the reviewer for this suggestion to make the introduction more specific. We have updated the introduction section to highlight the problem statement in more detail in lines 120-128. In the updated introduction section, we have also added a section to discuss the existing CDC SVI based studies. We believe these additions have helped highlight the proposed framework and socio-economic vulnerability index computation. We have also included the suggested literature in suitable sections and appreciate the suggestions.

Lines 120-128: "Most existing health risk studies treat the CDC's Social Vulnerability Index (SVI) as a standard baseline, often employing association-based analyses to understand the correlation between the SVI's parameters, or the overall SVI score itself, and various health hazards (Khazanchi et al., 2020; Nayak et al., 2020; Rygel et al., 2006; Tatar et al., 2023). While these studies highlight the usage of machine learning approaches in establishing relationships between these socio-economic parameters and infection rates, they often rely on a single, general SVI score or the parameters used in SVI computation to represent vulnerability to any health crisis. In contrast, our approach utilizes a machine learning method to establish a unique, risk-specific relationship for influenza infection by considering a unique set of socio-economic indicators."

While the authors acknowledge the economic burden of influenza, the model itself does not properly include crucial economic variables such as healthcare expenditure.

- We agree that healthcare expenditure and similar economic variables are crucial for a comprehensive understanding of influenza burden. However, our model's development was constrained by the availability of uniform and reliable state-level data across all our chosen sources for the 2022 season.

  While we did not include direct healthcare expenditure, our model does incorporate other socio-economic factors, such as median household income and poverty rates, which serve as proxy indicators for a community's economic status and ability to absorb the financial shock of a severe influenza season.

  We acknowledge this data limitation in our manuscript's discussion in lines 395-398 and emphasize that future research should aim to integrate more direct economic variables as they become available at a consistent level of aggregation. We believe that despite this constraint, our model provides a robust and valuable assessment of socio-economic vulnerability.

  "Nevertheless, future extensions of this work could benefit from exploring spatial or multilevel modeling frameworks, especially with finer-resolution data (e.g., county-level or census-tract level) and more variables (e.g. healthcare expenditure)."

Given the presence of a powerful and well-defined index such as the SVI, the authors need to better justify their choice of variables.

- We agree that the CDC's Social Vulnerability Index (SVI) is a powerful and well-defined tool for all-hazards preparedness, providing an excellent initial understanding of a community's vulnerability to a wide range of threats. However, our study focuses on the specific context of influenza-like infections, which have a unique set of drivers.

  Our variable selection was designed to go beyond the general-purpose nature of the SVI to create a threat-specific model. While the SVI uses a fixed set of 16 variables grouped into four broad themes, our approach identified and weighted 22 specific socio-economic indicators that are most directly associated with influenza infection rates. For example, our model includes variables like access to healthcare, which are particularly relevant to the spread of infectious diseases.

  We have provided detailed justification for our variable selection in the manuscript. Supplementary Table 1, along with our discussion of multicollinearity, shows that we selected variables to avoid redundancy and ensure that the final set of indicators is a robust and highly predictive set for understanding vulnerability to influenza. This specialized approach allows for a more precise and actionable assessment than a general-purpose index.

Although an attempt was made to explain the rationale for variable selection, some choices may not be defensible. For example, “No computer” may not be a strong indicator, as most individuals in the U.S. have access to cell phones, which arguably provide even greater access to information for the general population.

We thank the reviewer for this suggestion. The variables selection is briefly discussed in the main manuscript, but a detailed list of reasons is in Table S1 with appropriate references. We understand that due to a technical error, the supplementary information was not visible to the reviewers in the initial submission.

With regards to the specific example pointed out by the reviewer of the "No Computer", variable derived from the U.S. Census Bureau's American Community Survey (ACS), is a measure of a household's lack of access to a wide range of devices. The Census definition for this variable includes not only desktops or laptops, smartphones, tablets, and other portable wireless computers (*ACS Table S2801, "Types of Computers and Internet Subscriptions*). Therefore, a household categorized as having "no computer" is one that lacks all these devices, including a cell phone or smartphone. Given this definition, "No Computer" serves as a valid indicator of a household's limited access to digital information, which is an important factor in public health communication during an epidemic.

The discussion section lacks an in-depth analysis of how socioeconomic factors influence the results.

We appreciate the feedback. Following the suggestion, we have updated the discussion section to provide more in-depth analysis regarding the important socio-economic factors on vulnerability computation. Line 352-365:

“The ordered feature importance provides a data-driven roadmap for strategic public health and socio-economic policy intervention. Since the indicators are ranked by their influence on ILI vulnerability, government resources can be optimally directed toward the highest-leverage areas. Specifically, the strong importance of indicators related to health insurance coverage suggests that expanding access to affordable healthcare is necessary. High feature importance linked to the elderly, female, and African American populations necessitates specific, targeted public health programs to address the underlying social determinants that drive this age, gender, and race-specific risk, thereby working to close documented health disparity gaps. The results underscore the need for tailored public health measures, including expanded healthcare access in rural regions, targeted vaccination campaigns, improved insurance coverage, and initiatives addressing gender-specific healthcare disparities. By leveraging spatial vulnerability maps, policymakers can prioritize high-risk areas, allocate resources effectively, and develop proactive strategies to mitigate ILI risks. Stakeholders can implement effective measures to achieve the maximum reduction in state-level susceptibility to severe infectious disease outcomes, by prioritizing the top-ranked structural and demographic deficiencies.”

Have the authors made all data and (if applicable) computational code underlying the findings in their manuscript fully available? The PLOS Data policy requires authors to make all data and code underlying the findings described in their manuscript fully available without restriction, with rare exception (please refer to the Data Availability Statement in the manuscript PDF file). The data and code should be provided as part of the manuscript or its supporting information or deposited to a public repository. For example, in addition to summary statistics, the data points behind means, medians and variance measures should be available. If there are restrictions on publicly sharing data or code —e.g. participant privacy or use of data from a third party—those must be specified.

Reviewer #1: Yes

Reviewer #2: Yes

Reviewer #3: None

PLOS authors have the option to publish the peer review history of their article (what does this mean?). If published, this will include your full peer review and any attached files.

If you choose "no", your identity will remain anonymous, but your review may still be made public.

Do you want your identity to be public for this peer review? For information about this choice, including consent withdrawal, please see our Privacy Policy.

Reviewer #1: No

Reviewer #2: No

Reviewer #3: No

Figure resubmission:

While revising your submission, we strongly recommend that you use PLOS's NAAS tool (https://ngplosjournals.pagemajik.ai/artanalysis) to test your figure files. NAAS can convert your figure files to the TIFF file type and meet basic requirements (such as print size, resolution),

or provide you with a report on issues that do not meet our requirements and that NAAS cannot fix.

After uploading your figures to PLOS's NAAS tool - https://ngplosjournals.pagemajik.ai/artanalysis, NAAS will process the files provided and display the results in the "Uploaded Files" section of the page as the processing is complete. If the uploaded figures meet our requirements (or NAAS is able to fix the files to meet our requirements), the figure will be marked as "fixed" above. If NAAS is unable to fix the files, a red "failed" label will appear above. When NAAS has confirmed that the figure files meet our requirements, please download the file via the download option, and include these NAAS processed figure files when submitting your revised manuscript.

Reproducibility:

To enhance the reproducibility of your results, we recommend that authors of applicable studies deposit laboratory protocols in protocols.io, where a protocol can be assigned its own identifier (DOI) such that it can be cited independently in the future. Additionally, PLOS ONE offers an option to publish peer-reviewed clinical study protocols. Read more information on sharing protocols at https://plos.org/protocols?utm_medium=editorial-email&utm_source=authorletters&utm_campaign=protocols

References:

CDC. (2022, September 15). *Centers for Disease Control and Prevention/ Agency for Toxic Substances and Disease Registry/ Geospatial Research, Analysis, and Services Program. CDC/ATSDR Social Vulnerability Index 2022 Database US. https://www.atsdr.cdc.gov/placeandhealth/svi/data_documentation_download.html Accessed on 09/15/2025*.

Gu, T., Li, J., Wang, M., Duan, P., Zhang, Y., & Cheng, L. (2023). Study on landslide susceptibility mapping with different factor screening methods and random forest models. *PLoS ONE*, *18*(10 October). https://doi.org/10.1371/journal.pone.0292897

Hu, X., Wu, S., Zhang, G., Zheng, W., Liu, C., He, C., Liu, Z., Guo, X., & Zhang, H. (2021). Landslide displacement prediction using kinematics-based random forests method: A case study in Jinping Reservoir Area, China. *Engineering Geology*, *283*, 105975. https://doi.org/https://doi.org/10.1016/j.enggeo.2020.105975

Khazanchi, R., Beiter, E. R., Gondi, S., Beckman, A. L., Bilinski, A., & Ganguli, I. (2020). County-Level Association of Social Vulnerability with COVID-19 Cases and Deaths in the USA. In *Journal of General Internal Medicine* (Vol. 35, Issue 9, pp. 2784–2787). Springer. https://doi.org/10.1007/s11606-020-05882-3

Li, Z., & Demir, I. (2023). U-net-based semantic classification for flood extent extraction using SAR imagery and GEE platform: A case study for 2019 central US flooding. *Science of The Total Environment*, *869*, 161757. https://doi.org/https://doi.org/10.1016/j.scitotenv.2023.161757

Nayak, A., Islam, S. J., Mehta, A., Ko, Y.-A., Patel, S. A., Goyal, A., Sullivan, S., Lewis, T. T., Vaccarino, V., Morris, A. A., & Quyyumi, A. A. (2020). Impact of Social Vulnerability on COVID-19 Incidence and Outcomes in the United States. *MedRxiv : The Preprint Server for Health Sciences*. https://doi.org/10.1101/2020.04.10.20060962

Rygel, L., O'Sullivan, D., & Yarnal, B. (2006). A method for constructing a social vulnerability index: An application to hurricane storm surges in a developed country. *Mitigation and Adaptation Strategies for Global Change*, *11*(3), 741–764. https://doi.org/10.1007/s11027-006-0265-6

Strobl, C., Boulesteix, A.-L., Kneib, T., Augustin, T., & Zeileis, A. (2008). Conditional variable importance for random forests. *BMC Bioinformatics*, *9*(1), 307. https://doi.org/10.1186/1471-2105-9-307

Tatar, M., Faraji, M. R., & Wilson, F. A. (2023). Social vulnerability and initial COVID-19 community spread in the US South: A machine learning approach. *BMJ Health and Care Informatics*, *30*(1). https://doi.org/10.1136/bmjhci-2022-100703
